# 1H-NMR metabolomics-based surrogates to impute common clinical risk factors and endpoints

**DOI:** 10.1016/j.ebiom.2021.103764

**Published:** 2021-12-20

**Authors:** D. Bizzarri, M.J.T. Reinders, M. Beekman, P.E. Slagboom, BBMRI-NL, E.B. van den Akker

**Affiliations:** a Molecular Epidemiology, LUMC, Leiden, the Netherlands; b Leiden Computational Biology Center, LUMC, Leiden, the Netherlands; c Delft Bioinformatics Lab, TU Delft, Delft, the Netherlands; d Max Planck Institute for the Biology of Ageing, Cologne, Germany; e BBMRI-NL: https://www.bbmri.nl; see Consortium Banner Supplement S1

**Keywords:** BMI, Body Mass Index, med, medication (e.g. lipid or blood pressure lowering medication, hsCRP, high-sensitivity C-Reactive Protein, eGFR, estimated Glomerular Filtration Rate, chol, cholesterol, hgb, haemoglobin, wbc, white blood cells, metabolic surrogates, posterior probability obtained applying the models, MetaboWAS, metabolome-wide association studies, T2D, Type 2 Diabetes status, ^1^H-NMR metabolomics, Missing values, Epidemiology, Regression models, Association studies, Surrogate clinical variables

## Abstract

**Background:**

Missing or incomplete phenotypic information can severely deteriorate the statistical power in epidemiological studies. High-throughput quantification of small-molecules in bio-samples, i.e. ‘metabolomics’, is steadily gaining popularity, as it is highly informative for various phenotypical characteristics. Here we aim to leverage metabolomics to impute missing data in clinical variables routinely assessed in large epidemiological and clinical studies.

**Methods:**

To this end, we have employed ∼26,000 ^1^H-NMR metabolomics samples from 28 Dutch cohorts collected within the BBMRI-NL consortium, to create 19 metabolomics-based predictors for clinical variables, including diabetes status (AUC_5-Fold CV_ = 0·94) and lipid medication usage (AUC_5-Fold CV_ = 0·90).

**Findings:**

Subsequent application in independent cohorts confirmed that our metabolomics-based predictors can indeed be used to impute a wide array of missing clinical variables from a single metabolomics data resource. In addition, application highlighted the potential use of our predictors to explore the effects of totally unobserved confounders in omics association studies. Finally, we show that our predictors can be used to explore risk factor profiles contributing to mortality in older participants.

**Interpretation:**

To conclude, we provide ^1^H-NMR metabolomics-based models to impute clinical variables routinely assessed in epidemiological studies and illustrate their merit in scenarios when phenotypic variables are partially incomplete or totally unobserved.

**Funding:**

BBMRI-NL, X-omics, VOILA, Medical Delta and the Dutch Research Council (NWO-VENI).

**Research in context**

**Evidence before this study**

Blood metabolites are the downstream products of biological processes in our body, integrating the environmental influences as well as the host's genetic background, and potentially offering a holistic view of an individual's health status. In previous studies, high-throughput quantification of metabolite abundances in blood (metabolomics) have been shown to convey information regarding a wide array of phenotypic aspects (i.e., age, BMI), and clinical endpoints (i.e., type 2 diabetes, all-cause mortality). However, what is currently missing, is a systematic evaluation of the potentiality of the blood metabolome to impute common clinical variables in an epidemiological setting, and to assess whether truly independent information is captured with respect to clinical endpoints.

**Added value of this study**

Our study demonstrates that ^1^H-NMR metabolomics concentrations, coupled with machine learning algorithms, can reliably encode for a broad range of clinical variables that are routinely assessed in cohorts of BBMRI-nl, a consortium composed of ∼26,000 samples from 28 biobanks. We developed and examined metabolomics-based multivariate models for 20 different binary clinical variables, comprising physiological and body composition measures, environmental exposures, inflammatory factors, medication usage, blood cell composition, lipids metabolism and clinical endpoints.

**Implications of all the available evidence**

Although prior research has identified some methods to impute missingness, these findings suggest that metabolomics profiling has added value for providing faithful markers that can replace relevant missing information. This additional data can be used to complement the one already available in a cohort, but also to explore totally unobserved confounders in omics association studies. Finally, we observed that the newly obtained metabolic biomarkers present significant associations with all-cause mortality in older populations, stimulating further exploration of metabolomics-based risk factors profiles.

Alt-text: Unlabelled box

## Introduction

A major goal in biomedical research is to find faithful biomarkers of health, defined as accurate and reproducible assays that provide objective indications on the health of an individual and his/her risk of developing a disease over predefined time trajectory.^1^ Over the years, many types of putative biomarkers have been proposed, ranging from environmental factors to biochemical assays, that may aid the diagnosis and prognostication of disease, including cardiovascular disease, cancer, and immunological disorders. Many of these clinical variables, however, are costly or cumbersome to obtain, especially for more critical and frail participants, such as older individuals.^1^,^2^ Consequently, missing data frequently occurs in large epidemiological or clinical studies, potentially leading to a significant loss of statistical power, thus impeding biomarker research in studies of older individuals.^3^

Missing datapoints in a single phenotypic variable can be handled in various ways. Often, analyses are either restricted to individuals or variables with a complete dataset, which may introduce potential biases.^4^ Alternatively, missing observations can be imputed using other phenotypic variables that have been successfully collected for those samples. However, this approach works only satisfactorily if the complete phenotypic variables are informative for the ones with missing observations.^3^^,^^5^, ^6^, ^7^ A third solution basically extends the second approach by leveraging informative omics data to impute missing values in phenotypic variables. Particularly useful in this context are metabolites quantified in minimally invasive biomaterials, such as urine, saliva or blood plasma, obtained with proton Nuclear Magnetic Resonance (^1^H-NMR) assays.^8^ Although this technique only captures a modest number of analytes, ^1^H-NMR metabolomics data is frequently acquired in large-scale epidemiological studies, as it is a cost-efficient and reproducible data resource. The underlying motivation is that metabolite concentrations in blood seem to be direct readouts of various biological processes, incorporating cues of the environment as well as the host's genetic background, and hence may be regarded as intermediate phenotypes. Indeed, metabolomics has been shown to capture information on the effect of drug treatments,^9^ disease status,^10^, ^11^, ^12^, ^13^ functional and cognitive decline,^14^ and ageing.^15^,^16^ In addition, several studies used the blood metabolome to predict single anthropometric measures, i.e. BMI,^17^ or other physiological characteristics, i.e. sex^18^ or age.^15^ However, it remains unclear whether the blood metabolome captured by ^1^H-NMR could represent phenotypic information over a wider set of conventional clinical variables.

We hypothesize that a single set of blood metabolic markers combined in multiple algorithms may represent a range of conventional clinical variables. As a proof of concept, we generated metabolic surrogates for 20 variables of general clinical and epidemiological interest available in at least 6 of the cohorts collaborating in BBMRI-NL. Here we will designate these as conventional clinical variables and they comprehend physiological measures (sex, age, blood pressure, etc.), environmental exposures (current smoking, etc.), body composition measures (BMI, etc.), inflammatory factors (hsCRP), medication usage (lipids medication, etc.), blood composition (white cell counts, etc.) lipids metabolism (LDL-cholesterol, etc.), and cardiometabolic clinical endpoints (diabetes and metabolic syndrome). Acquiring data for all these variables is costly and requires sufficient biomaterial, meaning that not every study has collected the same set of phenotypic variables. We further explored these methods to establish metabolic surrogate values in the Leiden Longevity Study, which we used to showcase possible applications in epidemiological research. We showed the validity of the surrogates in an external cohort comparing them to the original values, we examined their association to further clinically valuable cardiometabolic health markers, and explored whether the metabolic surrogates associate, separately or combined, to all-cause mortality.

## Methods

### Study populations

The samples used for the current study are part of the BBMRI-NL Consortium (Dutch Biobanking and BioMolecular resources and Research Infrastructure, https://www.bbmri.nl/), which includes the following 28 Dutch biobanks: ALPHAOMEGA, BIOMARCS, CHARM, CHECK, CODAM, CSF, DMS, DZS_WF, ERF, FUNCTGENOMICS, GARP, HELIUS, HOF, LIFELINES, LLS_PARTOFFS, LLS_SIBS, MRS, NESDA, PROSPER, RAAK, RS, STABILITEIT, STEMI_GIPS-III, TACTICS, TOMAAT, UCORBIO, VUMC_ADC, VUNTR. A description of the cohorts included is provided in the Supplementary Materials S3. Ethics committees approved the protocols for these studies in all the involved institutes, and all participants provided informed consent. The whole data set contains samples of ∼31,000 individuals before quality control.

### Metabolomic measurements

The present study included metabolite concentrations measured in EDTA plasma samples using the high-throughput proton Nuclear Magnetic Resonance (^1^H-NMR) metabolomics (Brainshake Ltd./Nightingale Health©, Helsinki, Finland). This device provides the quantification of routine lipids, lipoprotein subclasses, fatty acid composition and various low-molecular weight metabolites including amino acids, ketone bodies, and glycolysis-related metabolites in molar concentration units. Details about the methods and applications of the NMR platform have been provided previously.^20^,^21^ A total of 226 metabolic features are reported for each EDTA plasma sample, including the ratios and derived measurements. However, to avoid overfitting we considered for our models only a subset of 63 features, which were previously selected to confer a mutually independent subset.^15^,^16^ This subset comprises the total lipid concentrations, fatty acids composition and low-molecular-weight metabolites including ketone bodies, glycolysis-related metabolites, amino-acids, and metabolites related to immunity and fluid balance (see Table S1 for the full list of metabolites). A more detailed description of the measured entities have been provided elsewhere.^20^,^21^

### Data Pre-processing

#### Pre-processing of metabolomics data

We included in our analyses all the cohorts reporting on all the 63 metabolic biomarkers, therefore we omitted CODAM (N = 254) and VUNTR (N = 3,896), which are missing acetoacetate and glutamine, respectively. We also decided to not consider the metabolites with low detection rates in more than one cohort (3-hydroxybutyrate) or which frequently failed to reach the minimum detection threshold (XL_VLDL_L, XXL_VLDL_L, L_VLDL_L, XL_HDL_L, L_HDL_L). We removed outlier samples with 1 or more missing metabolic measure (232 removed samples), 1 or more zeroes per sample (74 removed samples) and samples with any metabolite concentration level more than 5 standard deviations away from the overall mean per metabolomic variable (604 removed samples). The remaining 551 missing values in the dataset (0⋅037% of the values) were imputed using the function nipals of the R package pcaMethods,^22^ and we z-scaled the metabolic measures across all samples to have comparable concentration levels between metabolites. The final data matrix comprised 25,867 samples across 56 metabolic variables. For more details, see Supplementary Materials S2. The number of samples used to train a predictor for a clinical variable depended on the number of samples missing this phenotypic information (Table 1). More information about the range of each phenotype within each biobank can be found in the Supplementary Materials S2.

**Table 1:** Performances of the 20 metabolic predictors

Binary Outcomes (threshold)	#samples	#cohorts	# True positives	Results (AUC)	
				5-Fold CV(mean)	LOBOV(weighted mean)
Low eGFR (eGFR≤ 60 ml/min) ^34^	21,439	23	1,196 (5·6%)	0·99 [0·98-0·99]	0·97 [0·91-0·99]
High triglycerides (trig≥ 2.3 mmol/L) ^26^	13,401	11	1,645 (12·3%)	0·97 [0·97-0·98]	0·95 [0·84-0·99]
High LDL cholesterol (LDL ≥ 4.1 mmol/L) ^26^	13,261	11	2,051 (15·5%)	0·96 [0·96-0·97]	0·97 [0·86-0·98]
High total cholesterol (totchol ≥ 6.2 mmol/L) ^26^	16,586	11	3,206 (19·3%)	0·96 [0·96-0·96]	0·96 [0·83-0·99]
Low HDL cholesterol (HDL ≤ 1.3 mmol/L) ^26^	16,506	11	7,414 (44·9%)	0·95 [0·95-0·96]	0·95 [0·85-0·96]
Diabetes (TRUE/FALSE)	18,841	16	4,034 (21·4%)	0·94 [0·93-0·9]	0·86 [0·72-0·98]
Metabolic syndrome (TRUE/FALSE)	7,811	6	3,452 (44·2%)	0·93 [0·92-0·94]	0·86 [0·71-0·93]
Sex (male) (TRUE/FALSE)	21,610	23	10,281 (47·6%)	0·92 [0·92-0·93]	0·91 [0·73-0·99]
Lipid medication (TRUE/FALSE)	17,707	14	5,783 (32·7%)	0·91 [0·90-0·91]	0·85 [0·77-0·94]
Low age (age < 45 y.o.)	21,519	23	3,353 (15·6%)	0·89 [0·88-0·90]	0·80 [0·55-0·85]
High hsCRP (hsCRP > 3mg/L) ^35^	5,180	8	1,548 (29·9%)	0·86 [0·84-0·86]	0·81 [0·7-0·86]
Blood pressure lowering medication (TRUE/FALSE)	15,832	13	7,234 (45·7%)	0·82 [0·81-0·83]	0·71 [0·51-0·84]
High age (age≥65 y.o.)	21,519	23	8,273 (38·4%)	0·82 [0·80-0·83]	0·73 [0·64-0·86]
Obesity status (BMI≥30 kg/$m^{2}$ and w.c. ≥ 102 cm [M] BMI≥30 kg/$m^{2}$ and w.c. ≥ 93 cm [F]) ^25^	19,322	18	3,135 (16·2%)	0·78 [0·75-0·80]	0·76 [0·69-0·81]
Low hemoglobin (hgb ≤ 6.67 mmol/L [M]; hgb ≤ 7.62 mmol/L [F]) ^27^	10,508	6	1,299 (12·4%)	0·76 [0·73-0·78]	0·72 [0·63-0·75]
Low white blood cells (wbc≤ 4.5x$10^{9}$ L) ^27^	9,496	6	818 (8·6%)	0·73 [0·69-0·76]	0·61 [0·5-0·71]
Current smoking (TRUE/FALSE)	21,662	23	8,276 (38·2%)	0·71 [0·70-0·72]	0·63 [0·48-0·78]
Alcohol consumption (TRUE/FALSE)	16,430	13	11,763 (71·6%)	0·71 [0·70-0·73]	0·60 [0·48-0·70]
Middle age (45 y.o. ≥Age < 65 y.o.)	21,519	23	9,893 (46·0%)	0·71 [0·70-0·72]	0·58 [0·50-0·69]
High pressure (systolic ≥ 140 mmHg and diastolic ≥ 90 mmHg) ^26^	17,509	12	7,765 (44·3%)	0·68 [0·66-0·69]	0.60 [0·52-0·76]

# samples: number of participants; # cohorts: the number of cohorts that we could use for training the models and for the evaluation using 5-Fold Cross Validation (5-Fold CV); # True Positives: the number of samples with original variable equal to TRUE; Results (AUC): 5-Fold CV= the mean AUCs of the 5-Fold CV and LOBOV= the mean AUCs of the Leave One Biobank Out Validation weighted based on the size of the testing biobank. [M]: male, or [F]: female specific criteria, eGFR=estimated glomerular filtration rate, w.c=waist circumference, hgb=haemoglobin, wbc=white blood cells.

#### Binarization of the clinical variables

To emphasize the relevant clinical conditions, we used clinical thresholds to obtain dichotomous variables out of the set of the available continuous risk factors, separating between “normal” and “at risk” levels for each risk factor (in Table 1 and in the Supplementary Materials S2).

#### Composed clinical variables

We chose to include some composed clinical variables: 1) LDL cholesterol, which was calculated using the Friedewald equation^23^ with the measured hdl cholesterol, triglycerides levels, and total cholesterol; 2) eGFR (estimated Glomerular Filtration Rate), which is a measure for the kidney filtration rate of an individual, was calculated using the creatinine-based CKD-EPI equation^24^; 3) obesity, which is a binary variable describing if a person is clinically obese or not that uses BMI, waist circumference, and sex based on the finding of Flint et al.^25^; 4) high pressure, a binary variables which defines high blood pressure by using systolic and diastolic blood pressure^26^; 5) low_hgb (low hemoglobin), which is a binary variable describing ‘at risk’ levels of hemoglobin by using hemoglobin and sex.^27^

#### Estimation of the metabolic surrogates

Each clinical variable is predicted by a penalized logistic regression model:

(1) $$\pi_{k}=\mathrm{Pr}\left(c_{k}=1|\;X=m\right)=\frac{1}{1+e^{-\left(\beta_{0}+\;\beta^{T}m\right)}}$$

in which $c_{k}$ is one of the *k* clinical variables ($c_{k}=\left\{0,1\right\}$), m the matrix with the values of the 56 metabolic features, $\beta^{T}$ the vector of regression coefficients, and $\beta_{0}$ the intercept. The probability of a sample having a value of 1 for a clinical variable ($\pi_{k}$), then results in the (predicted) ‘surrogate value’ for that clinical variable. The models are trained using the R package glmnet^28^ with L1 $\left({∥\beta ∥}_{1}\right)$ and L2 norm $\left({∥\beta ∥}_{2}^{2}\right)$ as regularization penalties to avoid overfitting. We employed two different training-evaluation procedures (each repeated 5 times): 1) a double 5-Fold-Cross-Validation (5-Fold CV); and 2) a Leave-One-Biobank-Out-Validation (LOBOV), which consists of holding out one of the biobanks while training on the remaining biobanks.^15^ During training the mixing parameter α was fixed to 0.5 (like previously done by other authors^29^, ^30^, ^31^) and the weight of the penalization (λ parameter) is optimized, which also determines the number of metabolites used for prediction (due to the L1 penalization). For more details see supplement.

### Metabolome wide association studies

We conducted Metabolome Wide Association Studies (MetaboWAS) using the middle-aged cohort of the Leiden Longevity Study (LLS-PARTOFFs, 2,307 individuals, median age at baseline = 59 years old). As metabolites distributions are often skewed, we first transformed all metabolite measurements using a rank inverse normalization (RIN). Applying a PCA on the LLS-PARTOFFs dataset revealed that the first 40 principal components explain 99% of the variance in the metabolites (**Fig. S7a**). Hence, the *p-value* of the MetaboWASs were Bonferroni corrected using 40 tests, i.e. a *p*-*value* designated significant when smaller than 0.00125 (0.05/40), following a similar procedure as in Ahola-Olli *et al*.^32^ We performed 5 different MetaboWASs.

### Associations of the metabolic surrogates to all-cause mortality

We used Cox proportional hazards models with follow-up time as the time scale, to test for associations between the metabolic surrogate measures and incident endpoints, i.e. all-cause-mortality in LLS-SIBS. We checked for associations adjusting for age and sex. To avoid bias due to familial correlations from pedigrees, we used robust standard errors (calculated with the Huber sandwich estimator) implemented in R coxph function. Considering that the population in LLS-SIBS has a different inclusion criterium for men (age > 89 years old) and women (age > 91 years old), we also evaluated associations separately in men and women. *P-values* were corrected using Benjamini Hochberg separately for each selection (all individuals, men and women) and considered significant the FDR < 0·05. To select potentially interesting metabolic surrogates, we used a stepwise procedure for the Cox regression models, corrected for sex and age. Starting from a model containing the full set of available variables, we removed or added an unselected metabolic surrogate at each round based on the improvement on the model calculated from the Akaike Information Criterion and considering the *p*-value of each variable included in the model.

### Ethics

The complete ethical statements for each cohort participating in BBMRI-nl are available in “Supplementary Materials S3: BBMRI-nl Consortium descriptions”

### Role of funders

BBMRI-NL contributed to the generation of the metabolomics data and the data sharing and computational resource infrastructure. Involved researchers were (partially) paid by X-omics, VOILA, Medical Delta and Dutch Research Council NWO-VENI. Funding sources had no role in the design of this study, and did not have any role during its execution, analyses, interpretation of the data, or decision to submit results.

## Results

### 1H-NMR metabolomics can be used to successfully predict 19 out of 20 clinical variables routinely measured in epidemiological and clinical studies

**Figure 1:**
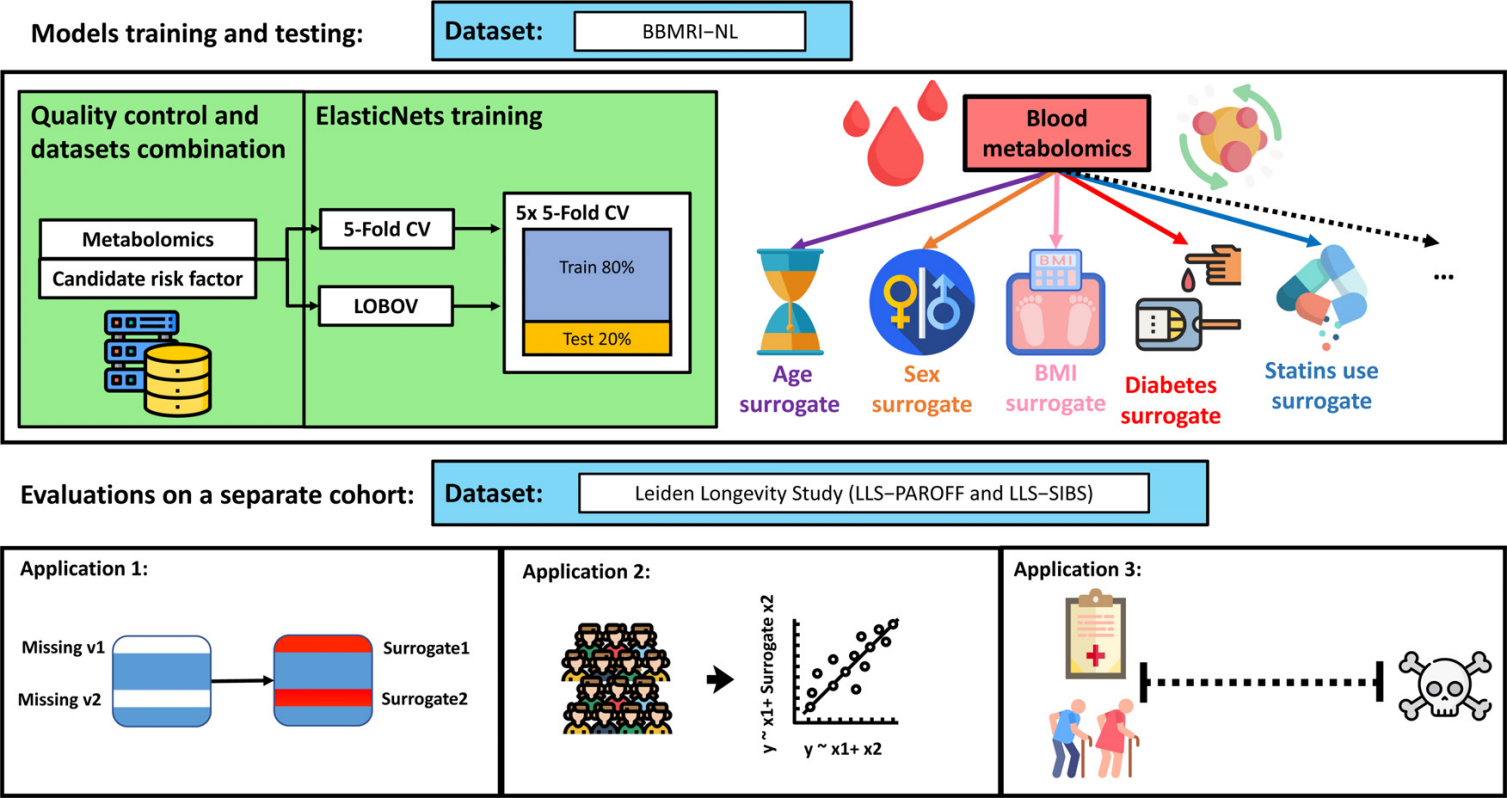

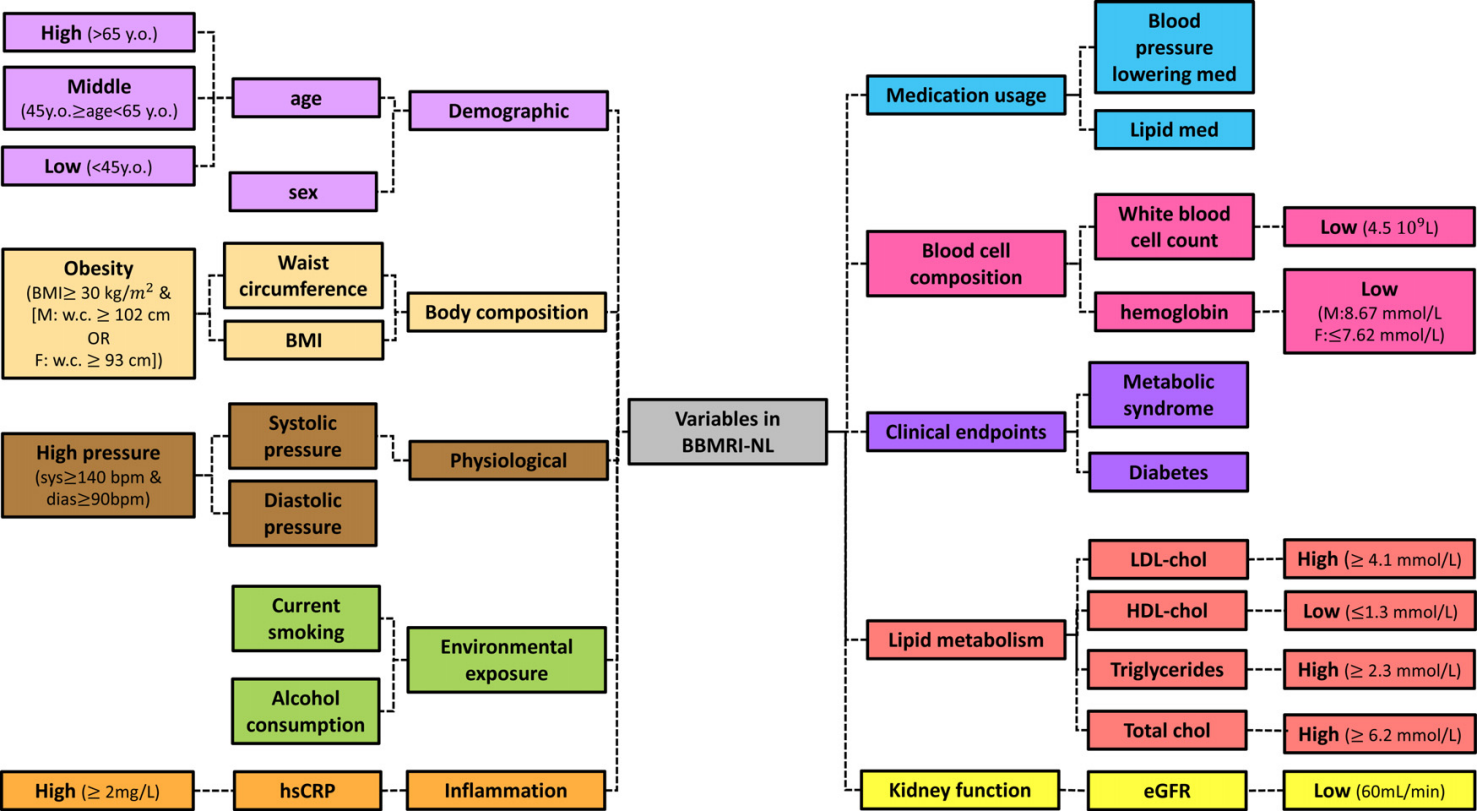
**Study design. [a]** Upper panel: Training of ^1^H-NMR metabolomics-based predictors for routinely assessed phenotypic variables available in BBMRI.nl. This data set was created as a collaboration of 28 community and hospital-based cohorts that collected nuclear magnetic resonance (^1^H-NMR) metabolomics data (Nightingale) for ∼31,000 individuals, before quality control. Upper panel left: Metabolomics-based predictors were trained using an *inner* loop of 5-fold Cross Validation (CV) (with 5 repetitions) for hyperparameter optimization and were evaluated in unseen data employing an *outer* loop of 5-fold CV or Leave-One-Biobank-Out-Validation (LOBOV). Upper panel right: using our models 19 different surrogate values can be derived from a single metabolomics data measurement to impute or complement a broad set of conventional clinical variables routinely assessed in epidemiological and clinical studies. Lower panel: Trained metabolomics-predictors were evaluated in two application scenarios using a held-out study, the Leiden Longevity Study.^19^ This study is a two-generation family-based cohort consisting of highly aged parents (LLS-SIBS, N = 817, median age = 92 years) and their middle-aged offspring and the partners thereof (LLS-PAROFF, N = 2,280, median age = 59 years), for which we had access to additional detailed phenotypic information. Trained predictors were evaluated for their ability to reconstruct missing datapoints in an independent dataset (Application 1, lower left), to be used as confounder in Metabolome Wide Association Studies (Application 2, lower central), and to investigate and to explore determinants of health in older individuals (Application 3, lower right). This image has been designed using resources from Flaticon.com. **[b]** Groupings of phenotypic variables routinely assessed in epidemiological and clinical studies for which data was available in BBMRI-NL. Continuous variables are dichotomized at levels generally accepted to confer an increased risk for cardio-metabolic endpoints. As various cutoffs on chronological age are in use, in part reflecting the highly non-linear relation between chronological age and disease risk, we choose to split chronological age in three categories (I ‘young’: < 45 years [TRUE/FALSE]; II ‘middle-aged’: ≥ 45 years [TRUE/FALSE]] and III ‘old’: < 65 years [TRUE/FALSE]]; ≥ 65 years). We integrated Body Mass Index, waist circumference and sex into one sex-specific measure of ‘obesity’. Similarly, we integrated diastolic blood pressure (DBP) and systolic blood pressure to arrive at one variable ‘high pressure’. Overall, we obtain data for 20 dichotomous phenotypic variables. Colors indicate groupings.

Missing or incomplete phenotypic information can severely deteriorate the statistical power in epidemiological studies. Here we evaluate the ability of Nuclear Magnetic Resonance (^1^H-NMR) metabolomics (Nightingale Health©, Helsinki, Finland) to reconstruct conventional clinical variables. For this purpose, we trained and evaluated prediction models (Figure 1**a**) for 20 conventional clinical variables (Figure 1**b**) using data of ∼26,000 individuals collected within the Dutch Biobanking and BioMolecular resources and Research Infrastructure (BBMRI-NL: https://www.bbmri.nl/). Out of 220 metabolomic variables measured on the platform, we employed 56 metabolic markers, selected to be the most uncorrelated^20^,^33^ and most successfully measured in the BBMRI studies (**Methods and Supplementary Materials S1**). Conventional clinical variables were transformed or constructed with the emphasis to be able to capture clinically relevant aspects of disease risk. For instance, we dichotomized continuous variables according to generally accepted clinical thresholds, thus obtaining for each of these clinical variables an ‘at risk’ [TRUE/FALSE] variable. For the same purpose, some variables were either merged or split. For instance, a sex-specific ‘*obesity*’ [TRUE/FALSE] variable was defined using body mass index, waist circumference, and sex, whereas chronological age was split into three categories **(**Figure 1**b)**. Each model outputs an uncalibrated posterior probability (Equation 1) that indicates the probability of a sample belonging to one of two labels. Overall, we were able to construct and evaluate 20 variables mainly representing risk factors of cardio-metabolic health that are routinely assessed in epidemiological and clinical studies.

Logistic Elastic-NET regression models were trained for each of the 20 dichotomous variables, measured in at least 6 of the BBMRI studies, in both healthy and diseased individuals. Model development was performed in two loops to prevent overtraining. An *inner* loop of 5-Fold Cross Validation with 5 repetitions was used to tune the hyperparameters of the model. Model performances where then evaluated in an *outer* loop of held out data, using again a 5-Fold CV or a Leave-One-Biobank-Out-Validation (LOBOV) (Figure 1**a, Methods**). We assessed model performances using the mean Area Under the Curve (AUC) of the receiver-operator curve obtained in the *outer* 5-Fold CV (Table 1) and considered a model's performance to be sufficiently accurate at AUC > 0·7.^34^ Overall, 19 out of 20 models passed this criterium, with only a single phenotypic variable, ‘*high-pressure*’, that could not be accurately captured by ^1^H-NMR (AUC_5-Fold CV_ = 0.68). Strikingly, 9 out of 20 models achieved an AUC_5-Fold CV_ > 0·9. While some of these high performances are expected as they directly relate to metabolic markers assessed on the platform (‘*Low eGFR*’, ‘*high triglycerides*’, ‘*high LDL cholesterol*’, ‘*high total cholesterol*’, and ‘*low LDL cholesterol*’), this is not the case for four other high performing models: ‘*diabetes*’ (AUC_5-Fold CV_ = 0·94), ‘*metabolic syndrome*’ (AUC_5-Fold CV_ = 0·93), ‘*sex*’ (AUC_5-FoldCV_ = 0·92), ‘*lipid medication*’ (AUC_5-Fold CV_ = 0·90). Also, other important cardio-metabolic health statuses, including ‘*obesity*’, ‘*high hsCRP*’, and ‘*blood pressure lowering medication*’ were predicted at a more than satisfactory accuracy (AUC_5-Fold CV_ > 0·8), indicating that overall, the ^1^H-NMR metabolome can be used to impute a broad spectrum of common clinical variables.

As the performances of our models may vary per biobank due to study-specific characteristics, e.g., varying study inclusion criteria or protocols for sample storage, we also evaluated the variation of our model performances across biobanks. First, using a Leave-One-Biobank-Out-Validation (LOBOV), we evaluate how our models would perform when applied to data of a new unseen biobank. As expected, mean model accuracies of the LOBOV, weighted based on the size of the testing biobank, show more variation across folds (**Figure S2a-b)** and are generally slightly lower than the overall results of the 5-Fold CV (Table 1). In particular, some of the smaller studies containing diseased patients showed relatively poor accuracies (**Figure S2b**). Indeed, surrogate values do show cohort specific effects, but interestingly, this does not necessarily affect its predictive performance within cohorts **(Figure S2c)**. Overall, 14 out of the 20 models performed on average satisfactorily (AUC_LOBOV_ > 0.7) across all studies in the LOBOV setting.

### Metabolic surrogates show dependencies mimicking the conventional clinical variables

**Figure 2:**
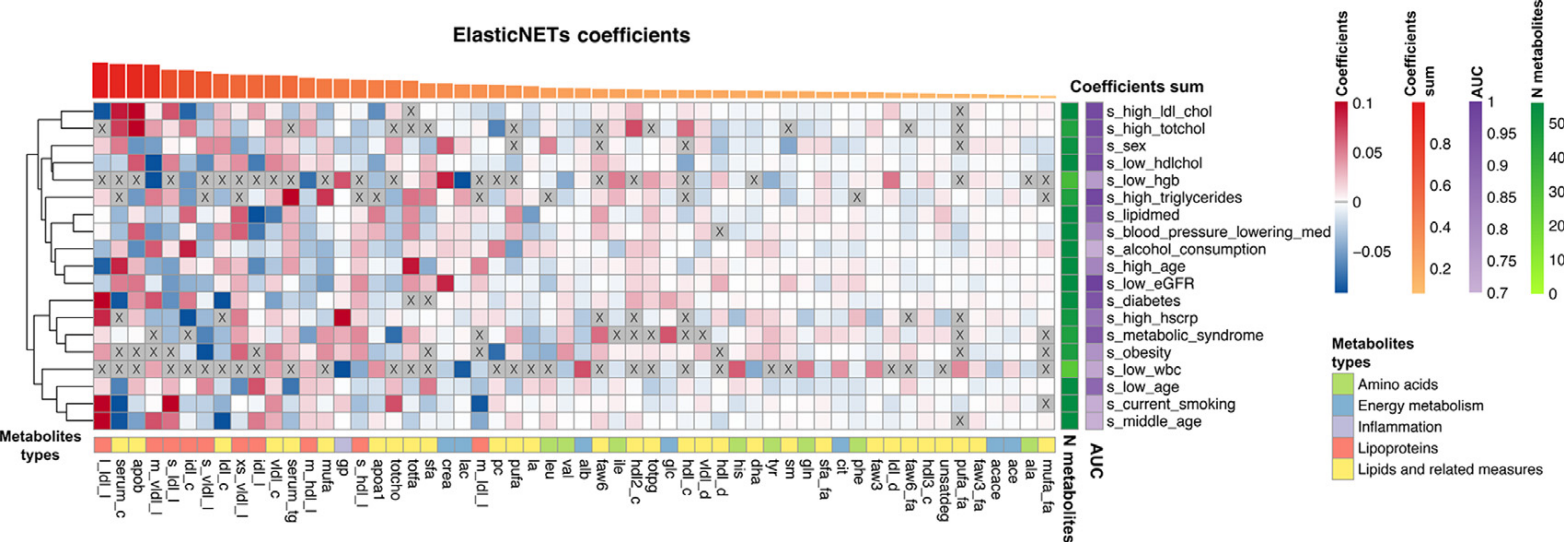
**ElasticNETs metabolites relative importance.** The heatmap reports the relative importance of the metabolites (columns) in each of the trained models (rows). Prior to visualization, metabolite coefficients were scaled per model by dividing them by the coefficients’ sum in each model to create the relative importance per model. Top: Metabolites were ordered based on the sum of their importance across all models. In addition, the models are clustered on the similarity between relative importance. Bottom: Categorized metabolic measures: “Amino acids”, “energy metabolism”, “inflammation”, “lipoproteins”, and “lipids and related measures”. Right: N. metabolites selected by the logistic ElasticNETs, Mean AUCs of the 5-FoldCV in a scale of purple.

Given that all models are trained on a relatively limited set of metabolic markers, we investigated to what extent the produced models and predictions show mutual dependencies. For this purpose, we first visualized the coefficients (betas) of the logistic Elastic-NETs (Figure 2) to show the relative importance of the metabolites within each of the prediction models. Only a few models select a limited number of metabolites (e.g., low wbc), while most of the models use a broad range of metabolites in different contributions (betas) to the prediction. However, these contributions still show some similarities across models, as quantified by the correlations between the model coefficients (**Figure S3**). Overall, we note a clear preference for the models to include metabolites of the classes “Lipoproteins” and “Lipids and related measures” over “Amino Acids”. In addition, we note that the models of related phenotypes also display some resemblances in the employed features, for instance ‘*lipid medication*’ and ‘*blood_pressure_lowering_medication’* share some model characteristics.

**Figure 3:**
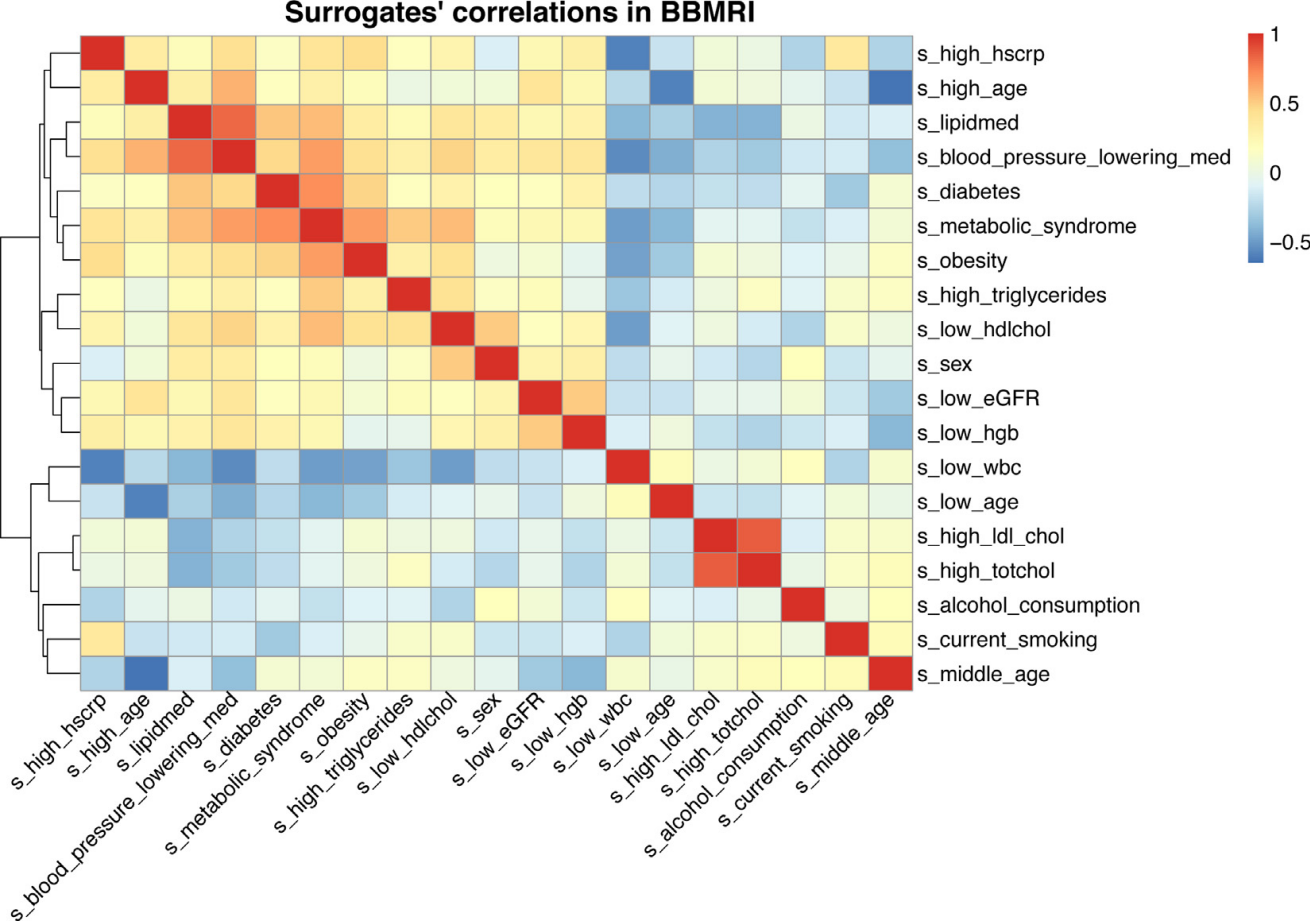
**Heatmap of pairwise correlations of the metabolic based surrogate markers calculated in BBMRI-NL.** The heatmap of correlations of the metabolic surrogate values of the 19 successful models, clustered based on the correlation levels, between the imputed metabolic surrogate levels within BBMRI-NL.

We next evaluated correlations between the outputs of our models, from here on referred to as the ‘metabolic surrogates’ (Figure 3**)** and compared these to correlations between the original clinical variables (**Figure S1b**) in the BBMRI.nl data set. Overall, we observe that the model outputs show correlation patterns and groupings that largely mimic that of the original variables. For instance, model outputs trained on variables related to weight problems, i.e. ‘*obesity*’, ‘*diabetes*’, ‘*metabolic syndrome*’, show high mutual correlations, and moreover are grouped with model outputs trained on medication usage, i.e. ‘*lipid medication*’ and ‘*blood pressure lowering medication*’. Although we observe some correlations between the outputs of our different age predictors e.g., ‘*low age*’, ‘*middle age*’, ’*high age*’, we observe that ‘*high age*’ is grouped with the models for ‘*high hscrp’*, ‘*lipid medication*’, and ‘*blood pressure lowering medication*’, while ‘*middle age*’ is grouped with ‘*current smoking*’ and ‘*alcohol use*’ and ‘*low age*’ with ‘*low white blood cell count’*. This suggests that at different ages, different conventional clinical variables play a role in physiology; an aspect well-known from literature.^36^, ^37^, ^38^ Overall, this indicates that our models show mutual dependencies similar to observations for the original clinical variables.

### Projection in an independent study demonstrates model accuracy

**Figure 4:**
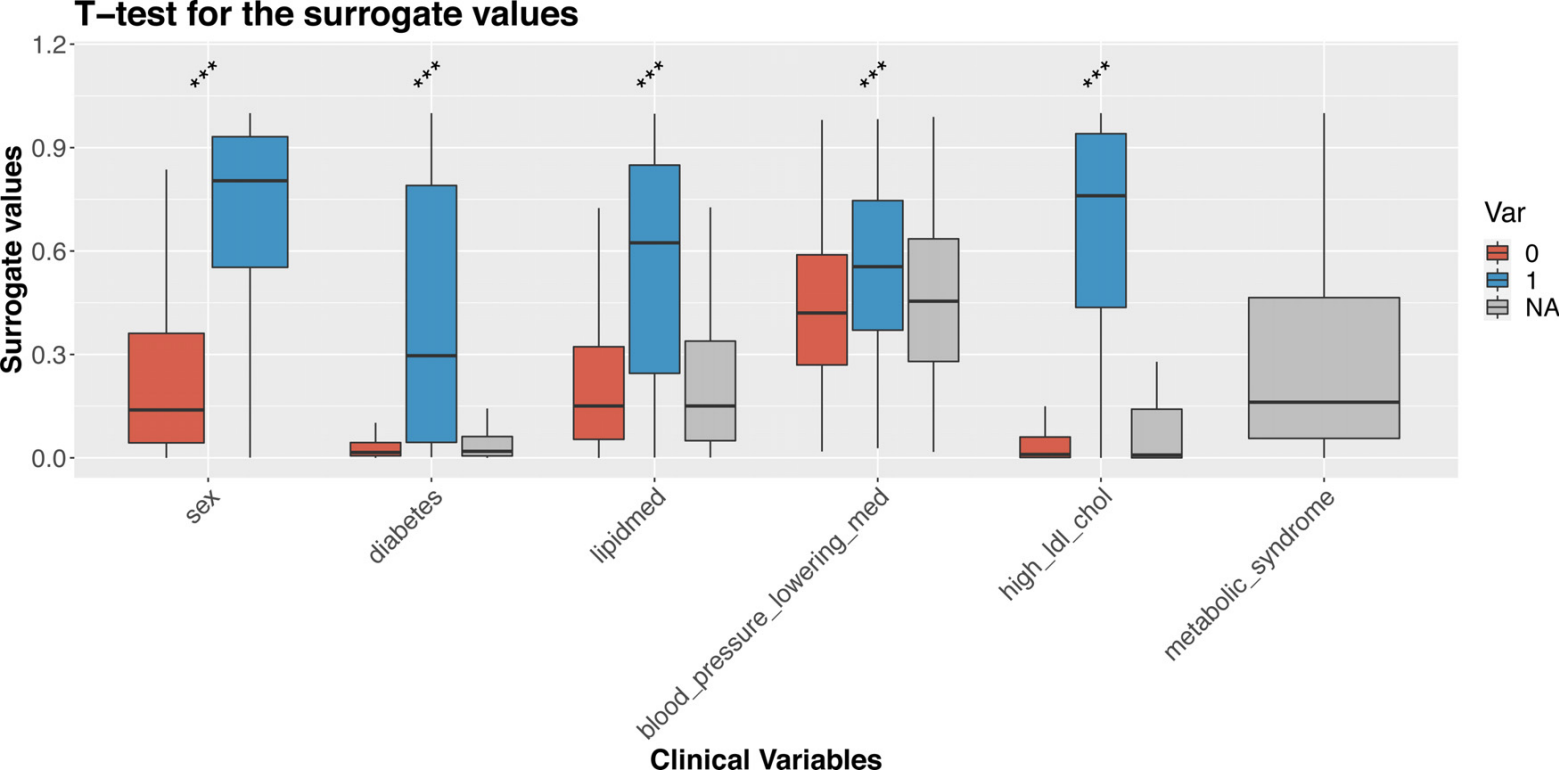
**Metabolic surrogates applied to LLS-PAROFF:** Paired boxplots show surrogate values split between the TRUE/FALSE (0/1) in the original values of the clinical variables (*** *p* ≤ 0.001 [t-test]). For metabolic syndrome the original variables are entirely missing, so no *p*-value is reported.

We performed a more extensive evaluation of the surrogate values by employing Nightingale ^1^H-NMR metabolomics and phenotypic data of the Leiden Longevity Study, a cohort excluded from the training and testing sets.^19^ The Leiden Longevity Study is a two-generation family-based cohort consisting of highly aged parents (LLS-SIBS, 851 individuals, age median = 92 years old) their middle-aged offspring and their partners (LLS-PAROFF, 2,307 individuals, age median = 59 years old). Using our models to project metabolic surrogates in the LLS-PAROFF gave an independent confirmation that conventional clinical variables can be readily captured by ^1^H-NMR metabolomics. Splitting the surrogate values by the actual labels of the corresponding binary phenotypes generally showed a good separation for important cardio-metabolic variables like ‘*sex*’, ‘*diabetes status*’, ‘*lipid medication’, ‘blood_pressure_ lowering_medication’,* and ‘*high LDL cholesterol*’ (Figure 4 and **S5**), emphasizing the suitability of our models for quality control purposes or to impute missing data. For instance, model results for ‘sex’ could be applied to verify absence of sample mix-ups (t.stat= 44·58, p=1·4 × 10^−313^ [t-test]). In addition, surrogate values seem informative on the nature of the missingness of phenotypic data. For instance, participants with a missing diabetes status typically had metabolic surrogate values similar to those of participants without diabetes (diabetes: $\mu_{F}$0=·05, $\mu_{T}$=0·41, $\mu_{NA}$=0·08), suggesting that a missing diabetes status generally implies ‘non-diabetics’ in this cohort. Similar observations were made for medication status (lipidmed: $\mu_{F}$=0·22, $\mu_{T}$=0·45, $\mu_{NA}$=0·21 and blood_pressure_lowering_med: $\mu_{F}$=0·44, $\mu_{T}$=0·55, $\mu_{NA}$=0·46): participants with missing statuses were more similar to non-medication users than medication users. In contrast, participants with missing values in LDL cholesterol had surrogate values indicating “at risk” levels of LDL cholesterol (high_ldl_chol: $\mu_{F}$=0·07, $\mu_{T}$=0·66, $\mu_{NA}$=0·14). Lastly, our surrogates also allow for explorative analyses of totally unrecorded variables. For instance, the ‘*metabolic syndrome*’ surrogate indicates participants who are more likely to have metabolic syndrome, a status which was not assessed in the LLS-PAROFF cohort (Figure 4**)**.

### Metabolic surrogates to explore confounders in Metabolome Wide Association Studies

We next explored the use of ^1^H-NMR metabolic surrogates to complement missing phenotypic data in metabolome-wide association studies (MetaboWAS). As an example, we evaluated the association of metabolic markers with Type 2 Diabetes status (T2D) in absence of information on a known potential confounder: BMI. We designed a controlled experiment to evaluate to what extent surrogate ‘obesity’ can replace BMI, using data of 1,697 individuals of LLS-PAROFF with complete metabolomic, BMI, and diabetes status, of which 79 are diagnosed with type 2 diabetes.

First, we ascertained that BMI was indeed a confounder, also within the LLS-PAROFF, by showing that BMI associated with the outcome (Type 2 Diabetes status, *t*-*test*= -7·83, *p* = 8·25 × 10^−15^ [t-test] **Figure S6a**), as well as many of the determinants (147 significant metabolites after correction, see **Methods**) of the MetaboWAS. Concomitantly, further adjustment of the MetaboWAS on T2D for BMI drastically reduced the number of significant metabolites. To compare, when adjusting for age and sex we identified 136 metabolites significantly associated with diabetes status, whereas further adjustment for BMI identified 80 significant metabolites (**Figure S7b Comparison 1**). Next, we performed the same association analyses using the ‘*obesity*’ surrogate as confounder. Similar to BMI, the ‘*obesity*’ surrogate is significantly higher in diabetics as compared to non-diabetics (*t*-*test* = -11·2, *p* = 2·48 × 10^−28^ [t-test] **Figure S7b**) and was associated with many of the metabolites (176 significant associations). Further adjusting the MetaboWAS on T2D for ‘*obesity*’ reduced the number of significant metabolites to 66 (**Figure S7b Comparison 2**).

**Figure 5:**
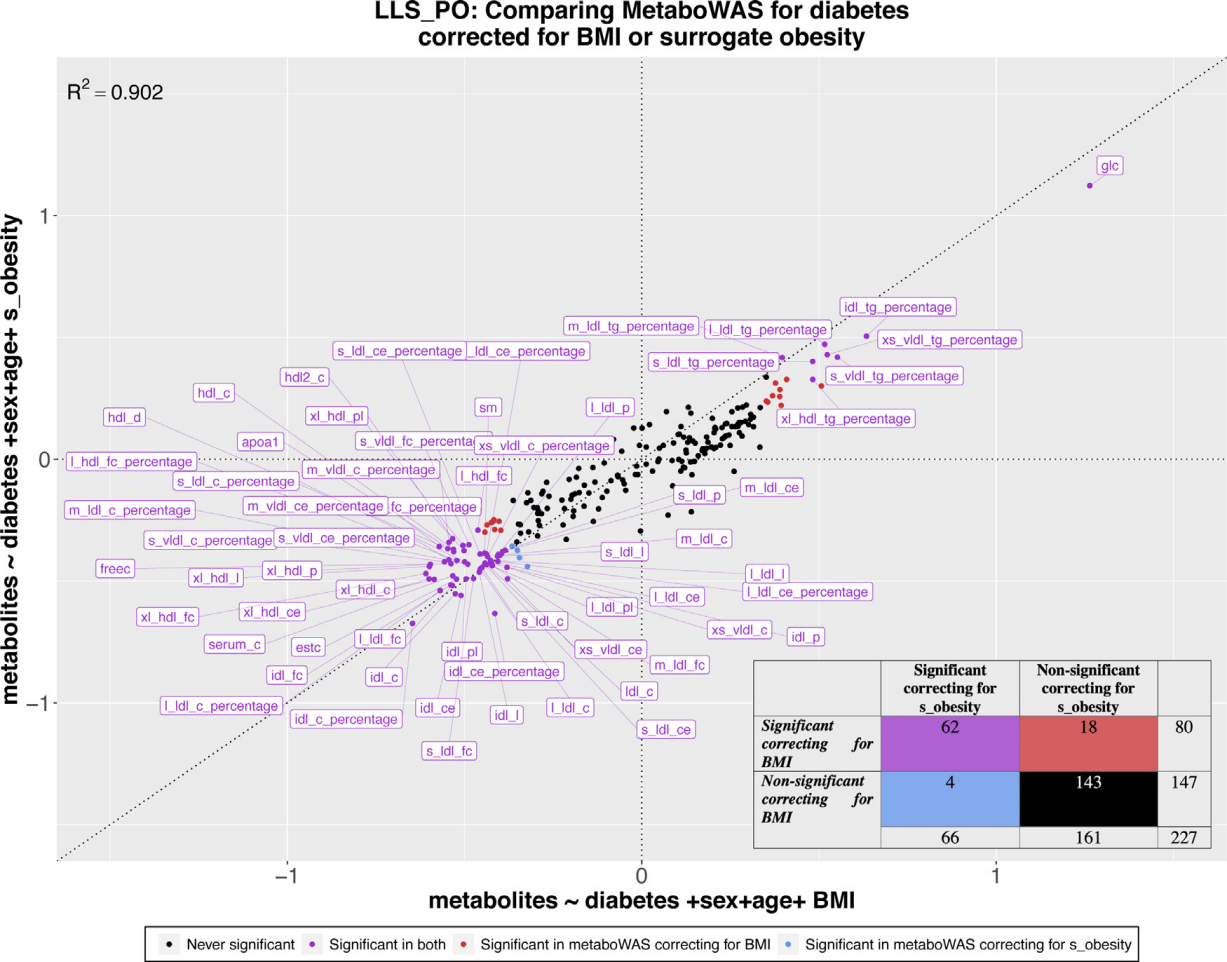
**Comparison between the estimated coefficients of metaboWAS on T2D adjusted for BMI or for surrogate *‘obesity’*.** On the x-axis the metaboWAS for diabetes adjusting for BMI and on the y-axis the metaboWAS for diabetes adjusted for surrogate obesity. The dataset composed of 1,697 individuals, 79 of which are diabetics. Estimated coefficients for each metabolite (points) are colored based on their significance in the two models: purple: significant in both; red: significant when adjusted for BMI only; blue: significant when adjusted for surrogate obesity only; black never significant. Lower right corner: a contingency table with the number of significant and non-significant metabolites identified using the two models.

We then investigated to what extent adjusting for BMI or adjusting for the ‘*obesity*’ surrogate yields similar metabolite markers to T2D associations, by comparing the obtained estimates from both models (Figure 5). Overall, highly similar (r^2^=0·902) associations between metabolic markers and T2D are found for both models, with glucose being the most significantly associated marker in both (p=9·43 × 10^−28^ [linear regression] when correcting for BMI and p=1·6 × 10^−26^ [linear regression] when correcting for *‘obesity’*). While most metabolites reported to be significantly associated with T2D overlap between the two models (62 out of 227; in purple), some discrepancies were observed, particularly at the significance threshold. When correcting for BMI, 18 significant metabolic markers were identified, that were not identified when correcting for ‘*obesity*’ (red dots, false negative rate ∼ 0·11). Conversely, 7 metabolites were deemed significantly associated with diabetes status when adjusting for ‘*obesity*’, but not when adjusting for BMI (blue dots, false positive rate ∼ 0·027). Nevertheless, overall, the differences in estimated effects remain small, indicating that metabolic surrogates may prove useful to account for missing data in epidemiological studies.

### Metabolic surrogates associate with all-cause mortality in older individuals

**Figure 6:**
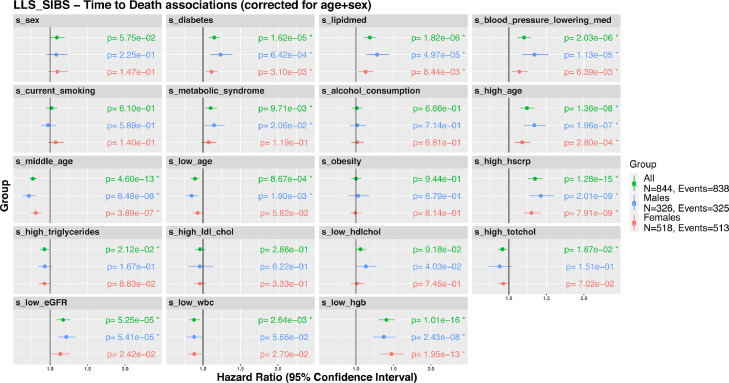
**Associations of metabolic surrogates with all-cause mortality.** Associations of the metabolic surrogates with time to all-cause mortality in LLS-SIBS, in groups comprising the entire set (“All”, N = 844 with 838 reported deaths), males (N = 326 with 325 reported deaths) or females (N = 518 with 513 reported deaths).

Next, we evaluated whether metabolic surrogates are indicative of health at old age, by associating these with all-cause mortality in a nonagenarian subsample of the Leiden Longevity Study (LLS_SIBS; 844 individuals, median age at baseline: 92 years old) (Figure 6**a**). Previous studies have already pointed out the ability of blood-metabolomics to predict or incident endpoints^39^ or all-cause^16^ mortality. In accordance, using Cox proportional hazards models adjusted for sex and age at inclusion for each of the 19 metabolic surrogates (**Methods**), we observed that 13 out of the 19 surrogates associated significantly with all-cause mortality (Figure 6**,** ‘all’). In line with previous reports, we observed the largest effect sizes with the surrogate levels of ‘*high age’*, ‘*medications usage’*, ‘*diabetes status’,* ‘*high hscrp’,* and ‘*hemoglobin’.* As previous studies have reported sex-specific associations for these clinical variables with all-cause mortality, we conducted a stratified analysis.^40^, ^41^, ^42^, ^43^, ^44^, ^45^, ^46^ Although, the direction of association with all-cause mortality remains generally the same between men and women, the strengths and their significance are in some cases different. For instance, the surrogate ‘*diabetes’* is associated with a higher risk on mortality in men (HR = 1·23, *p* = 6·42 × 10^−4^ [Cox regression]), than for women (HR = 1·11, *p* = 3·1 × 10^−3^ [Cox regression]), the same goes for ‘*blood pressure lowering medication’* (men: HR = 1·3, *p* = 1·13 × 10^−5^ [Cox regression], women: HR = 1·1, *p* = 6·39 × 10^−3^ [Cox regression]). In contrast, ‘*low hemoglobin*’ is associated with a higher risk in women (HR = 1·5, *p* = 1·95 × 10^−13^ [Cox regression]), than men (HR = 1·37, FDR = 2·42 ×  10^−8^ [Cox regression]).

**Figure 7:**
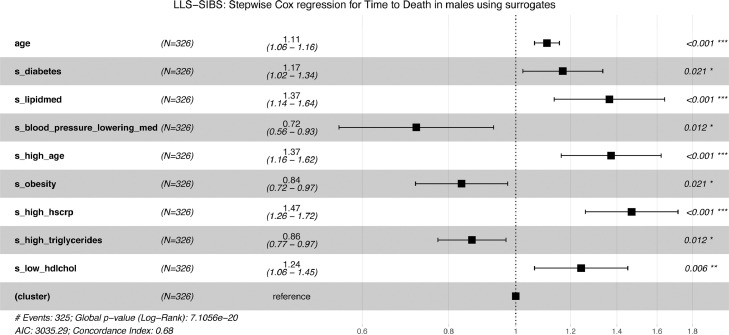

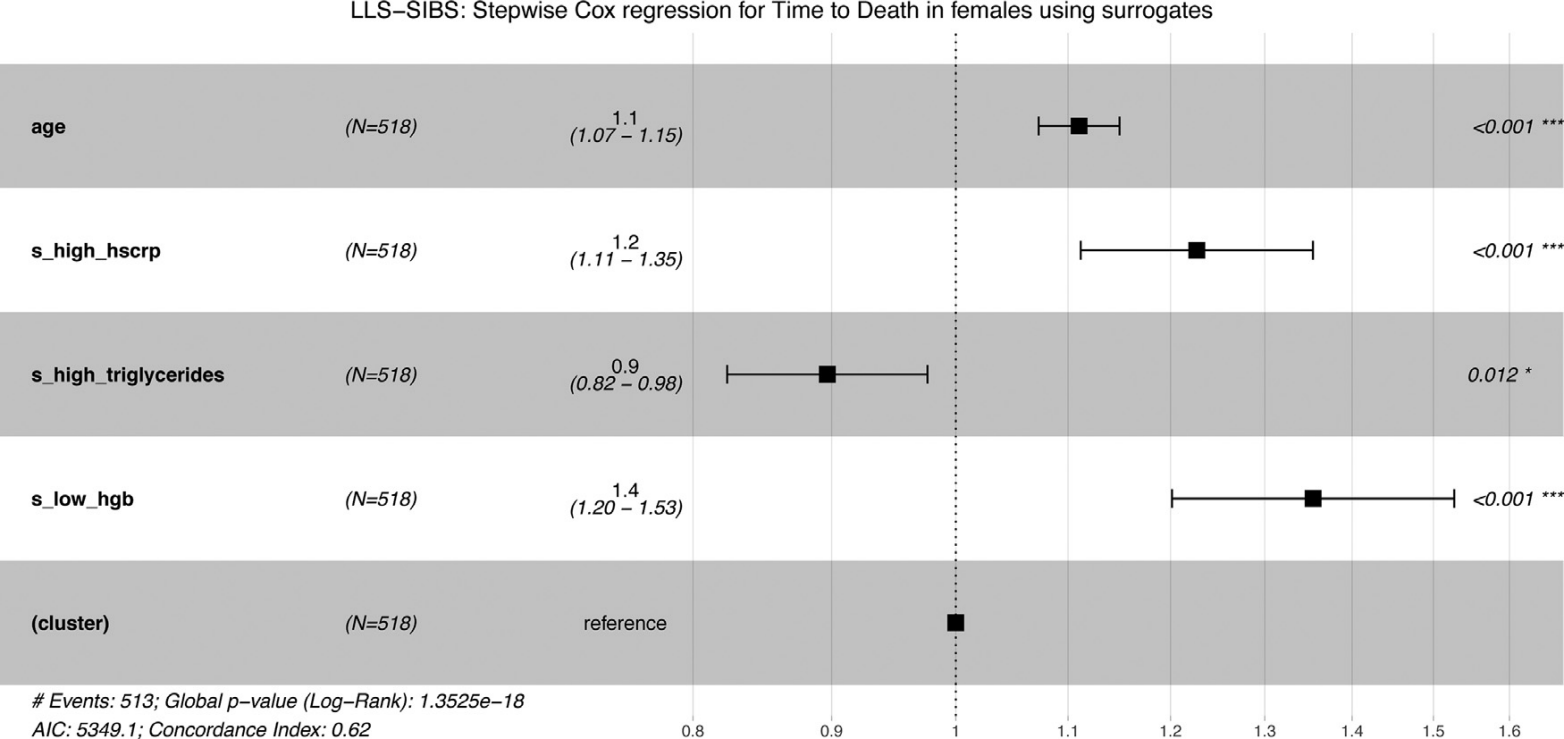
**Composite metabolomics predictors of all-cause mortality:** Predictors of time to death for males **[a]** and females **[b]**, in LLS-SIBS, composed using the surrogate metabolic measures, sex and age. “(cluster)” refers to the variable controlling for family relationships (methods)**.** Cox regression models were made using a step forward/backward selection.

To identify the minimal set of metabolic surrogates independently associating with all-cause mortality, we performed a stepwise (forward/backward) cox regression, adjusted for age at sampling, in the LLS-SIBS dataset (Figure 7**a-b**), stratified for sex. The surrogates ‘*high hsCRP*’ and ‘*high triglycerides*’ emerged as independent predictive features in both male and female models, associated with an increased and decreased risk respectively. While eight surrogates contributed to the male model, including ‘*lipid medication’*, ‘*high age*’, ‘*high hsCRP’,* and ‘*low hdlchol’*, only three surrogates contributed to the mortality prediction in females: ‘*high hsCRP’*, ‘*high triglycerides’,* and *‘low hemoglobin’*. These findings are in line with previous reports that different risk factors seem to predict survival up to the highest ages for the different sexes.^47^, ^48^, ^49^ However, we add a more extensive discussion upon the selection of ‘*high age*’ only in men in Supplementary Material 1 (document S3).

## Discussion

Missing phenotypic data is common in large epidemiological studies and in particular impedes biomarker research in older individuals. We employed ^1^H-NMR metabolomics data as a single source of information to successfully impute 19 out of 20 conventional clinical variables that mainly relate to cardio-metabolic health. We highlighted the potential of our imputation models for conventional clinical variables with three application scenarios. First, we applied our models to an independent study, the Leiden Longevity Study, demonstrating that we can reconstruct conventional clinical variables at high accuracy. Secondly, we showed the value of metabolic surrogates in omics studies when data on potential confounders is missing. Finally, we exemplified how metabolic surrogates can be used to explore risk factors of health in older individuals by showing that multiple metabolic surrogates are independently predictive of all-cause mortality.

Using logistic ElasticNET regression models we were able to reconstruct a broad range of conventional clinical variables pertaining to physiological measures, body composition measures, environmental exposures, inflammatory factors, medication usage blood cell composition, lipids metabolism, and clinical endpoints. For this purpose, we also constructed composite variables that may better capture particular aspects of health, for instance, our ‘*obesity*’ variable integrates body mass index, waist circumference, and sex to create a sex-specific measure for overweight. The selection was based on the most abundant variables within the 28 Dutch cohorts included in BBMRI-nl and considered as a good representation of the standardly collected variables in epidemiological studies. In the scope of the current paper, we considered a phenotypic dataset to be “incomplete” whenever it is missing one or more of those clinical variables considered as “commonly collected”, regardless of the main research question of the user.

We chose to construct binary representations of the continuous clinical variables for several reasons. First, we binarized continuous variables for a practical reason – to be able to judge all models on the same criteria. Secondly, predicting continuous variables using linear ElasticNET regression models is more prone to be affected by outliers, whereas the current approach emphasizes the prediction of the most commonly populated phenotypic range in which participants become at risk. Thirdly, our models output a posterior probability that indicates the probability (a continuous score) of a sample belonging to one of two labels, e.g. *obese*/*non-obese*. In effect, these posteriors reconstitute part of the information lost when dichotomizing continuous variables, as exemplified by the observed correlation patterns between surrogates that mimic the correlation patterns between the original variables.

Our pre-trained models for conventional clinical variables allow for the imputation of missing datapoints in partially incomplete phenotypic variables, and moreover they offer the opportunity to explore associations with completely unobserved phenotypic variables. The latter is very much in line with the current use of PolyGenic Scores (PGSs).^50^, ^51^, ^52^ A PGS captures the genetic propensity of the realization of a particular polygenic phenotype. Nearly a thousand PGSs have been collected,^53^ which can be used to systematically explore correlations between a measured variable of interest and a wide array of phenotypes-by-proxy in genetic studies. We propose a similar use for metabolic surrogates in large metabolomics studies, yet with two noteworthy distinctions. Whereas PGSs can arguably be used to tease out causality in so-called Mendelian Randomization studies,^54^ metabolomic surrogates cannot. In contrast, while PGSs often only explain a very modest part of their respective phenotypes, metabolic surrogates explain a much larger part, thus enabling different types of applications. We illustrated this in our second application scenario where we showcased the use of surrogates to explore potential confounding by non-assessed phenotypic variables in omics studies. While use of actual phenotypic variables will always be preferred over metabolic surrogates, the availability of these metabolic surrogates can thus be used to direct replication efforts or to inform the design of new or follow-up studies.

Besides anthropometric measures and other physiological characteristics, the blood ^1^H-NMR metabolome was previously also shown to capture aspects directly pertaining to health outcomes. In particular, we and others have previously reported ^1^H-NMR metabolomics-based risk estimators of cardiometabolic disease,^32^,^55^ pneumonia and COVID infection,^39^ and all-cause mortality.^16^ While this clearly illustrates the vast potential of the blood ^1^H-NMR metabolome as a universal readout for health outcomes, it also raises the question what factors give rise to metabolomic profiles associated with adverse outcomes. Given that the variation in the ^1^H-NMR metabolome is the result of a complex interplay of both environmental and genetic factors, we evaluated whether our surrogates might give us a first indication. To do so, we tested which of our surrogates might be indicative of all-cause mortality in an elderly subset of the LLS-study. Intriguingly, by employing our pre-computed models as well as when we built multi-variate cox-regression models for time-to-death, we find metabolic surrogates that relate to conventional clinical risk factors known to associate with mortality risk at old age. Moreover, sex-stratified analyses recapitulate some of the known differences in mortality associations observed at old age, with for instance many more risk factors independently associated for mortality in males, as compared to females. These results illustrate that metabolic surrogates can aid in the interpretation of metabolomics-based risk estimators.

This study has several limitations. LOBOV analyses revealed that the trained surrogates may show study-specific effects that may relate to employed procedures of data collection or sample storage of the cohorts under investigation. While these artifacts may be addressed using batch-correction algorithms,^56^ or employing deep learning models for the prediction tasks, we note that differences between studies may also be due to valid biological reasons, such as differences in inclusion criteria. These cohort-specific differences might also affect the prior probabilities regarding the clinical variable of interest, which we now assume to be constant (by assuming a fixed β_0_ in the model). Although logistic ElasticNET regression is not so sensitive to these differences in priors,^57^ one can also correct for these differences by adding a calibration step (e.g. Platt Calibration^58^). However, in order to do that, there needs to be partial information on the clinical variable with a representative prior distribution, which we often will not have when dealing with incomplete datasets. Another limitation resides in the population's characteristics, which may limit the potential generalizability of the results. Even though the models were trained in the large BBMRI-nl dataset, with a broad age range, this population is composed exclusively by Caucasians mostly healthy or with cardiovascular problems. Hence, care should be taken to generalize our surrogate models to intersection of populations that are not present in the training set, such as individuals of other ethnicities, younger than 18, or suffering from other diseases. Lastly, the number of biomarkers captured by the targeted NMR platform is small compared to the whole human metabolome (over 19,000 according to the Human Metabolome Database^59^). We acknowledge that more elaborate high throughput platforms (e.g., Mass Spectrometry) might better capture the aspects of some clinical variables, allowing to reach even higher accuracy levels. However, this often comes with less robust measurements, elevated additional costs and, as a result, reduced sample sizes. The latter introduces the danger of overfitting to the aforementioned study-related artifacts.

In conclusion, we have shown that the blood metabolome assayed by ^1^H-NMR metabolomics can successfully capture a broad set of conventional clinical variables opening various possibilities to exploit surrogates of these clinical variables in in large epidemiological and clinical studies.

## Declaration of interests

The authors declare that there are no competing interests.

## Appendix Supplementary materials

Image, application 1

Image, application 2

Image, application 3

Image, application 4
