# Supplementary material for: 1H-NMR metabolomics-based surrogates to impute common clinical risk factors and endpoints
Source: eBioMedicine. 2021 Dec 20;75:103764. doi: 10.1016/j.ebiom.2021.103764 (PMC8703237; doi:10.1016/j.ebiom.2021.103764)
Supplement: Supplementary file 1 [file mmc1.docx]

Supplementary Figures and Methods

This document contains the supplementary figures and methods for the paper "^1^H-NMR metabolomics-based surrogates to impute common clinical risk factors and endpoints”. The document is divided in 3 sections:

1. A section with supplementary figures, which we find important to understand some of the discussions in the paper.
2. A Supplementary Table containing a description of the metabolites used.
3. A section with supplementary Documents useful to better understand the methods used.

Table of Contents

[Supplementary Figures 2](#_Toc88837620)

[Figure S1: Clinical variables correlations in BBMRI 2](#_Toc88837621)

[Figure S2: Leave One Biobank Out Validation 3](#_Toc88837622)

[Figure S3: Correlation of the ElasticNETs’ coefficients: 4](#_Toc88837623)

[Figure S4: Missingness in LLS-SIBS and LLS_PARTOFFS 5](#_Toc88837624)

[Figure S5: T-tests of the surrogates in LLS Partner-Offspring 6](#_Toc88837625)

[Figure S6: T-test of the association of diabetes 8](#_Toc88837626)

[Figure S7: MetaboWAS of diabetes status corrected for BMI or surrogate obesity. 9](#_Toc88837627)

[Supplementary Tables 11](#_Toc88837628)

[Table S1. Description of metabolic variables used to build the predictors 11](#_Toc88837629)

[Supplemental Documents 13](#_Toc88837630)

[Document S1: Estimating the metabolic surrogates 13](#_Toc88837631)

[Document S2: On fixing the ElasticNET mixing parameter 15](#_Toc88837632)

[Document S3: Discussion upon the use of “](#_Toc88837633)*[high age](#_Toc88837633)*[” for the Stepwise Cox regression models. 18](#_Toc88837633)

[References 20](#_Toc88837634)

# Supplementary Figures

## Figure S1: Clinical variables correlations in BBMRI

[a]

[b]

**Figure S1: [**a] Heatmap representing the Jaccard indices calculated between each risk factors in BBMRI, [b] Correlation of the original clinical variables in BBMRI.

## Figure S2: Leave One Biobank Out Validation

[a]

[b]


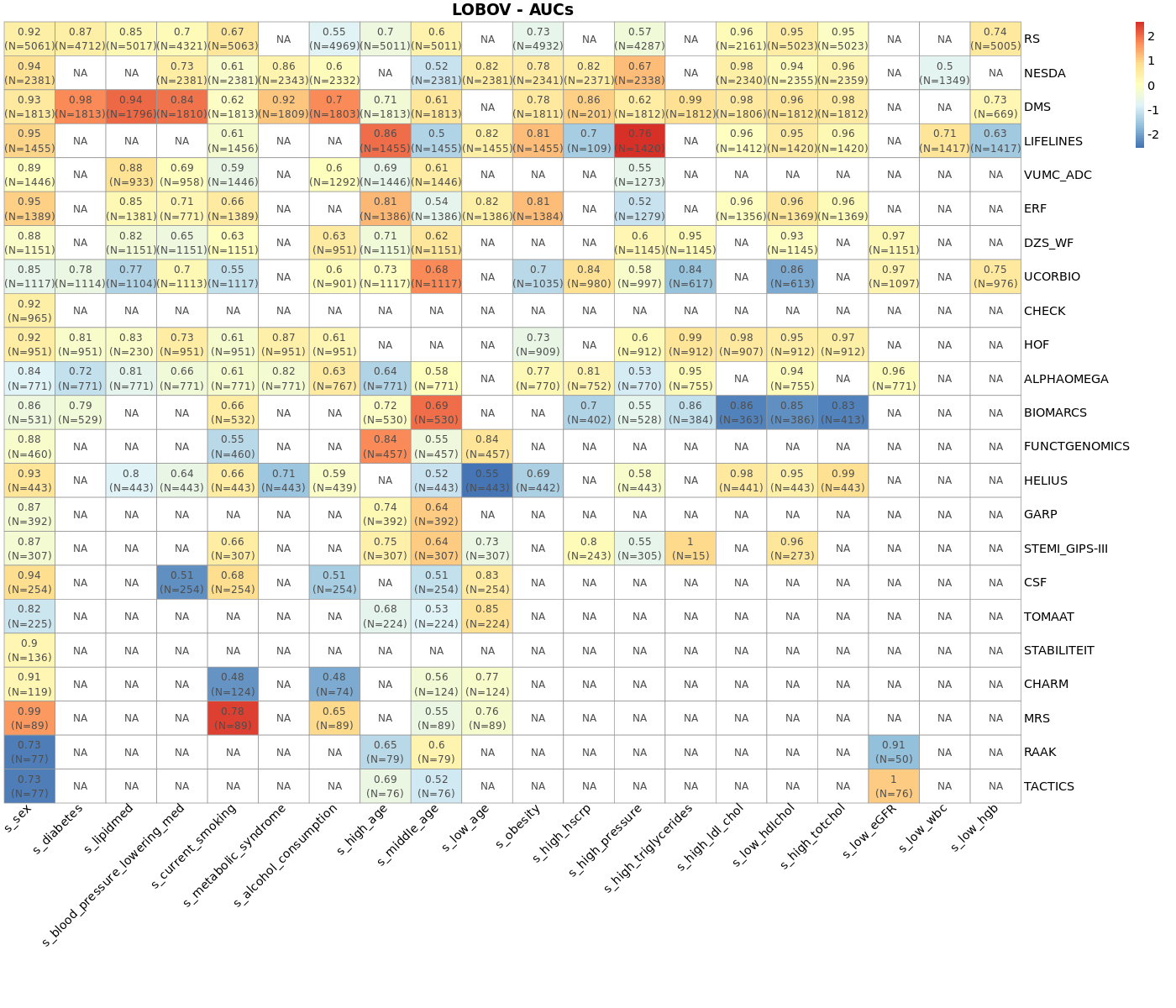


**Figure S2:** [a] The boxplots of the AUC obtained with LOBOV. The dots represent the AUC for each biobank, colored according to the percentage of one of the dichotomous values for the risk factor. [b] The heatmap of the AUC obtained in the LOBOV from each metabolic surrogate measure in each biobank. The AUCs are scaled based on the AUC for each metabolic model. Inside each cell we show also the available number of samples for each biobank.

## Figure S3: Correlation of the ElasticNETs’ coefficients:

**Figure S3:** Correlation heatmaps between the ElasticNETs’ betas of the final models.

## Figure S4: Missingness in LLS-SIBS and LLS_PARTOFFS

[a]

[b]


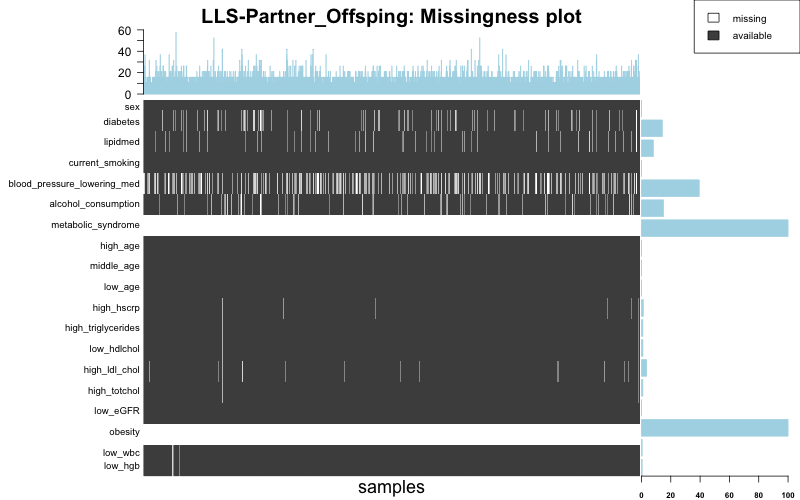


**Figure S4:** [a] Missing values in LLS-SIBS, [b] Missing values in LLS-PARTOFFS. The heatmaps show the status of a particular measurement (row), for a particular sample (columns). The bar plots on the sides indicate the percentage of missing measurement per clinical variable (on the right), or per sample (on the top).

## Figure S5: T-tests of the surrogates in LLS Partner-Offspring

**[a]**

| 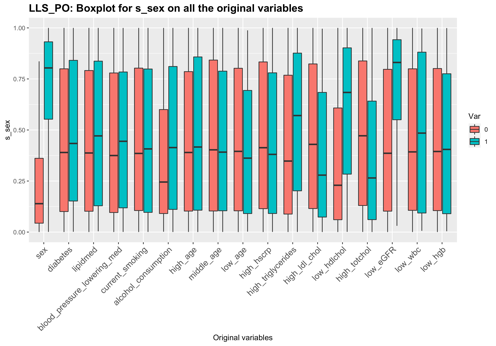 | 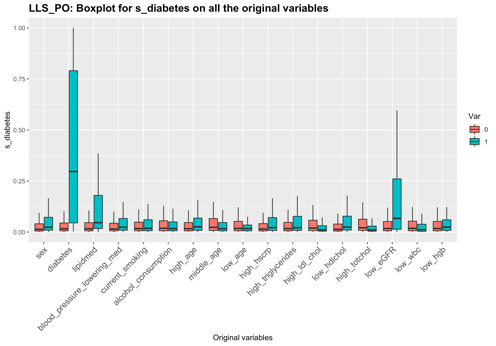 | 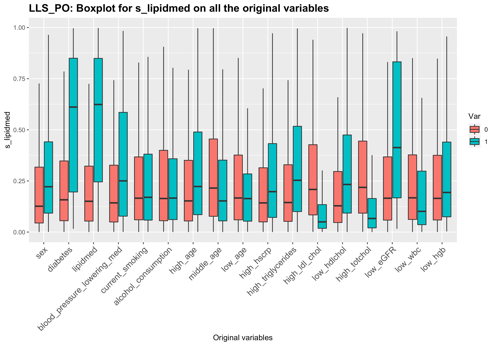 |
| --- | --- | --- |
| 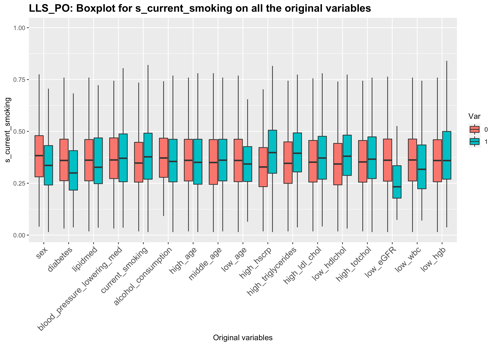 | 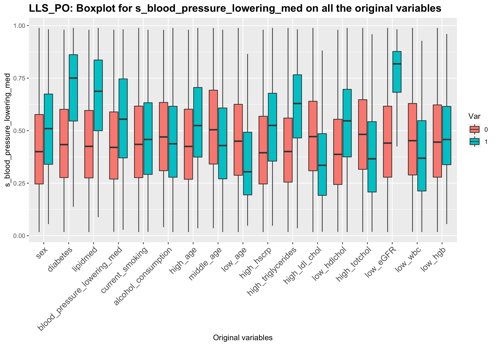 | 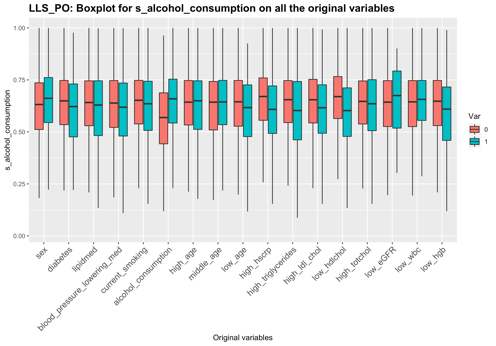 |
| 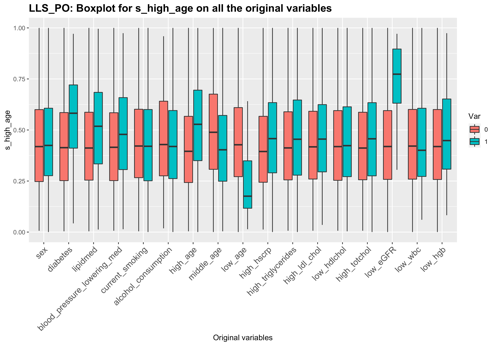 | 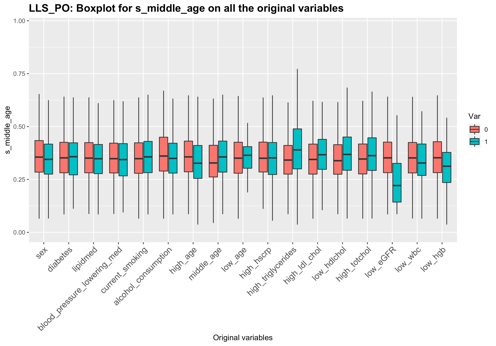 | 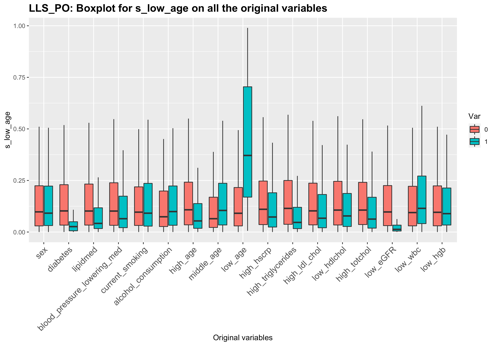 |
| 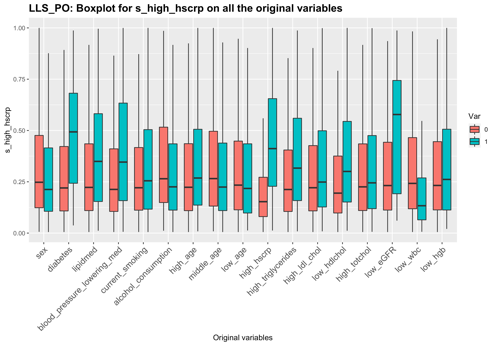 | 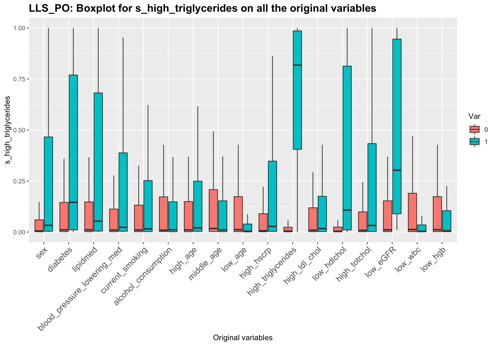 | 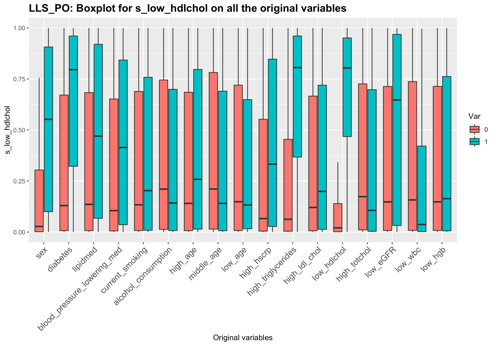 |
| 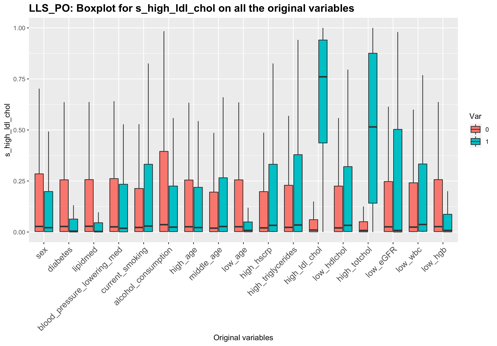 | 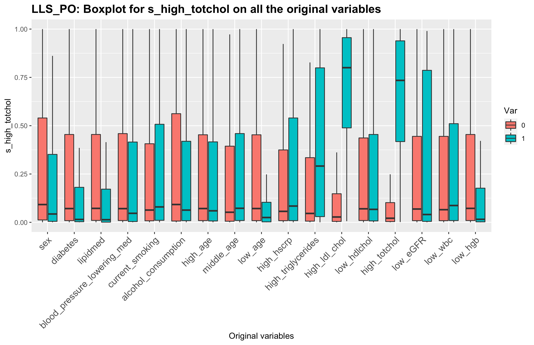 | 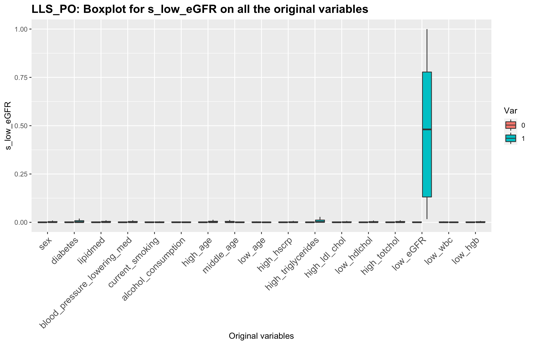 |
| 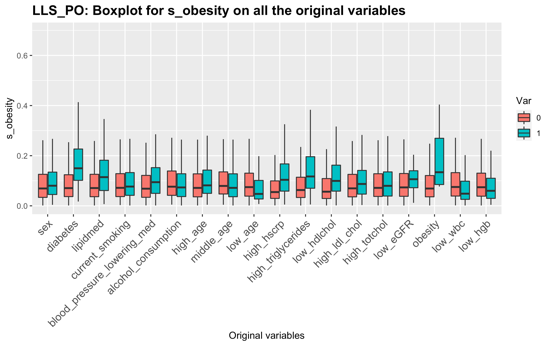 | 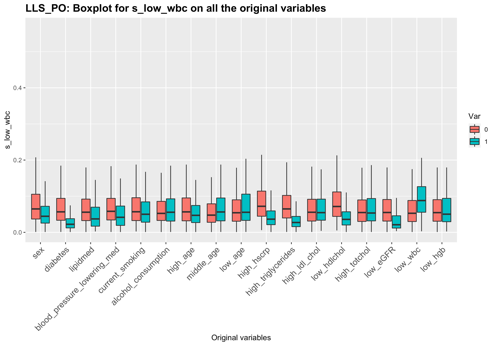 | 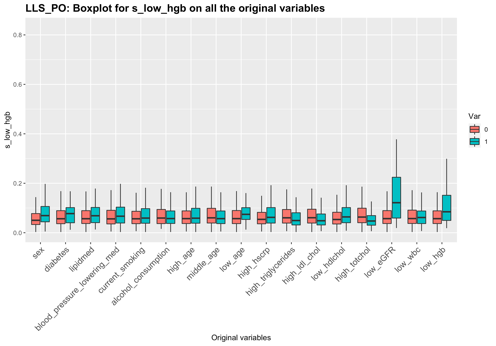 |

[b]

**Figure S5:** [a] Table reporting the results of the *t*-tests done on the surrogate markers to separate for each of the original variables. [b] Heatmap reporting all the *t*-statistics of the *t*-tests in Figure S5A. The numbers represent the *t*-statistics and there is an asterisk in case the test is significant (*p*-value < 0.05).

## Figure S6: T-test of the association of diabetes

**[a]**


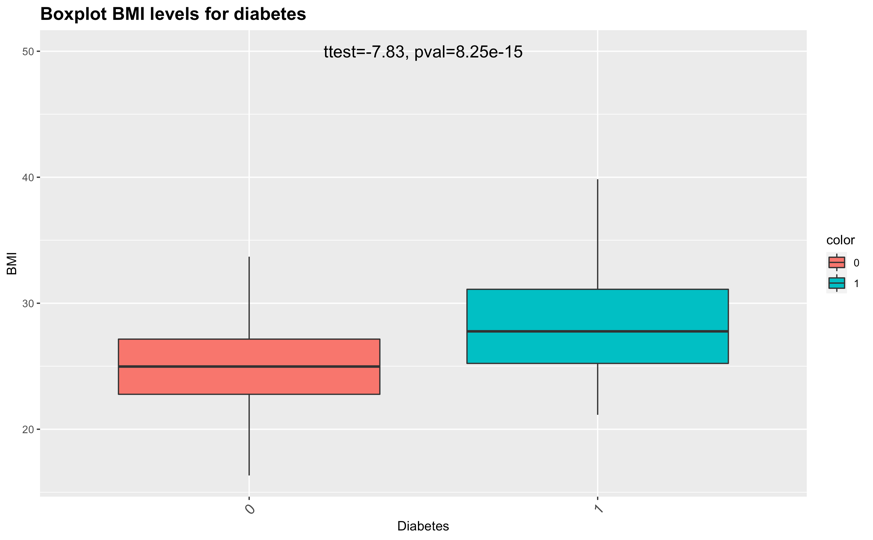


**[b]**


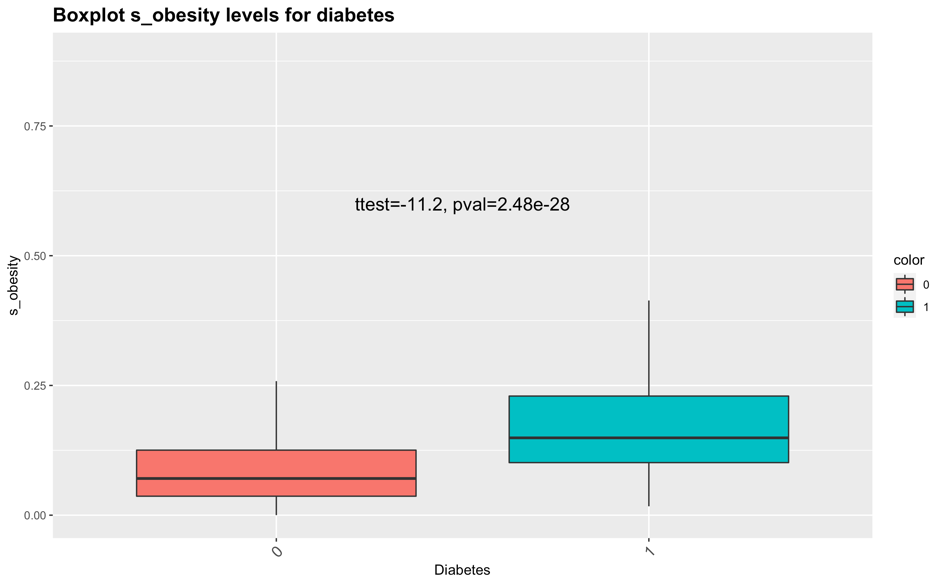


**Figure S6:** *T*-test of the association of diabetes and [a]) BMI or [b] s_obesity in LLS-PAROFF.

## Figure S7: MetaboWAS of diabetes status corrected for BMI or surrogate obesity.

**[a]**


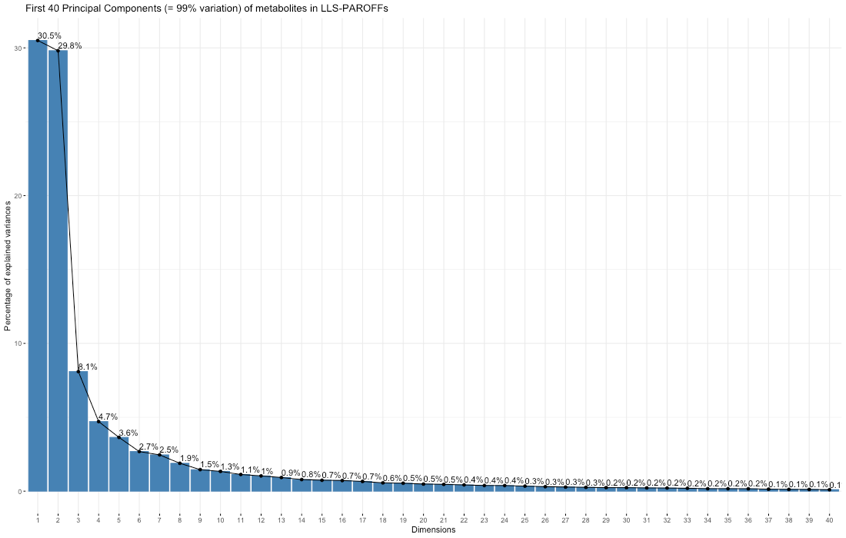


**[b]**

**[c]**

| **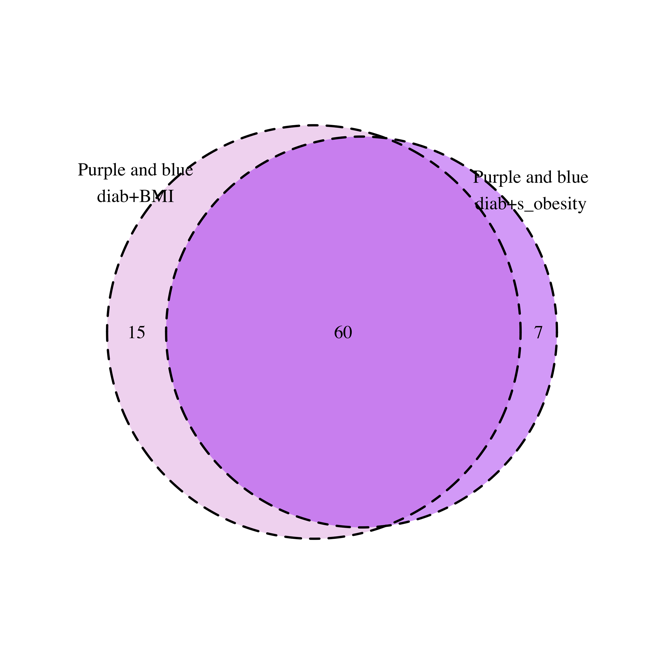** | **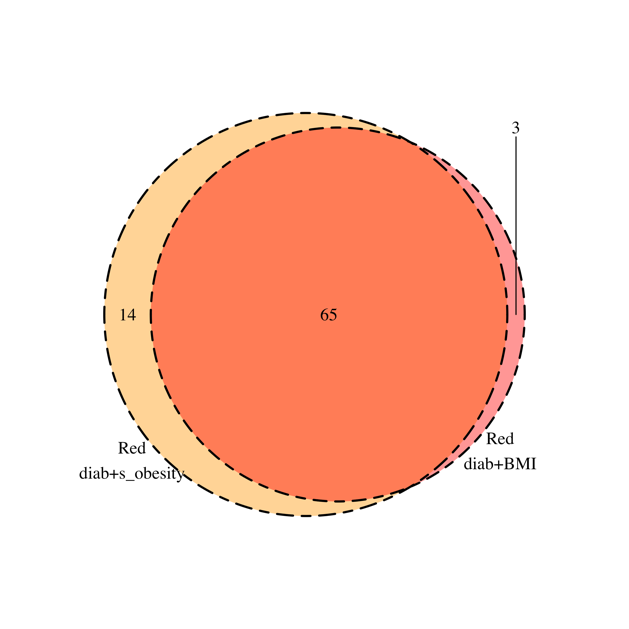** |
| --- | --- |
|  | |

**Figure S7:** [a] Scree-plot of the PCA conducted in the metabolic features in LLS_PAROFFs. [b] Comparison of the coefficient estimate of 3 metaboWAS. Comparison 1: The comparison of the metaboWAS for diabetes not corrected with the metaboWAS corrected for BMI; Comparison 2: The comparison of the metaboWAS for diabetes not corrected with the metaboWAS corrected for surrogate obesity; Comparison 3: The comparison of the metaboWAS for diabetes corrected for BMI with the metaboWAS corrected for surrogate obesity. [c] On the left the Venn diagrams that shows the interception of the metabolites that are not significant anymore when correcting for BMI and surrogate obesity; on the right the Venn diagrams that show the interception of the metabolites that become significant when correcting for BMI and surrogate obesity; finally under them the interception between the metabolites that stays significant when adding BMI or surrogate obesity, respectively.

# Supplementary Tables

## Table S1. Description of metabolic variables used to build the predictors

| **Metabolic variable** | **Description** | **Unit** |
| --- | --- | --- |
| **m_vlvl_l** | **Total lipids in medium VLDL** | **mmol/l** |
| **s_vldl_l** | **Total lipids in small VLDL** | **mmol/l** |
| **xs_vldl_l** | **Total lipids in very small VLDL** | **mmol/l** |
| **idl_l** | **Total lipids in IDL** | **mmol/l** |
| **idl_c** | **Total cholesterol in IDL** | **mmol/l** |
| **l_ldl_l** | **Total lipids in large LDL** | **mmol/l** |
| **m_ldl_l** | **Total lipids in medium LDL** | **mmol/l** |
| **m_hdl_l** | **Total lipids in medium HDL** | **mmol/l** |
| **s_hdl_l** | **Total lipids in small HDL** | **mmol/l** |
| **s_ldl_l** | **Total lipids in small LDL** | **mmol/l** |
| **vldl_d** | **Mean diameter for VLDL particles** | **nm** |
| **ldl_d** | **Mean diameter for LDL particles** | **nm** |
| **hdl_d** | **Mean diameter for HDL particles** | **nm** |
| **serum_c** | **Serum total cholesterol** | **mmol/l** |
| **vldl_c** | **Total cholesterol in VLDL** | **mmol/l** |
| **ldl_c** | **Total cholesterol in LDL** | **mmol/l** |
| **hdl_c** | **Total cholesterol in HDL** | **mmol/l** |
| **hdl2_c** | **Total cholesterol in HDL2** | **mmol/l** |
| **hdl3_c** | **Total cholesterol in HDL3** | **mmol/l** |
| **serum_tg** | **Serum total triglycerides** | **mmol/l** |
| **totpg** | **Total phosphoglycerides** | **mmol/l** |
| **pc** | **Phosphatidylcholine and other cholines** | **mmol/l** |
| **sm** | **Sphingomyelins** | **mmol/l** |
| **totcho** | **Total cholines** | **mmol/l** |
| **apoa1** | **Apolipoprotein A-I** | **g/l** |
| **apob** | **Apolipoprotein B** | **g/l** |
| **totfa** | **Total fatty acids** | **mmol/l** |
| **unsatdeg** | **Estimated degree of unsaturation** |  |
| **dha** | **22:6, docosahexaenoic acid** | **mmol/l** |
| **la** | **18:2, linoleic acid** | **mmol/l** |
| **faw3** | **Omega-3 fatty acids** | **mmol/l** |
| **faw6** | **Omega-6 fatty acids** | **mmol/l** |
| **pufa** | **Polyunsaturated fatty acids** | **mmol/l** |
| **mufa** | **Monounsaturated fatty acids; 16:1, 18:1** | **mmol/l** |
| **sfa** | **Saturated fatty acids** | **mmol/l** |
| **faw3fa** | **Ratio of omega-3 fatty acids to total fatty acids** | **%** |
| **faw6_fa** | **Ratio of omega-6 fatty acids to total fatty acids** | **%** |
| **pufa_fa** | **Ratio of polyunsaturated fatty acids to total fatty acids** | **%** |
| **Mufa_fa** | **Ratio of monounsaturated fatty acids to total fatty acids** | **%** |
| **sfa_fa** | **Ratio of saturated fatty acids to total fatty acids** | **%** |
| **glc** | **Glucose** | **mmol/l** |
| **lac** | **Lactate** | **mmol/l** |
| **cit** | **Citrate** | **mmol/l** |
| **ala** | **Alanine** | **mmol/l** |
| **gln** | **Glutamine** | **mmol/l** |
| **his** | **Histidine** | **mmol/l** |
| **ile** | **Isoleucine** | **mmol/l** |
| **leu** | **Leucine** | **mmol/l** |
| **val** | **Valine** | **mmol/l** |
| **phe** | **Phenylalanine** | **mmol/l** |
| **tyr** | **Tyrosine** | **mmol/l** |
| **ace** | **Acetate** | **mmol/l** |
| **acace** | **Acetoacetate** | **mmol/l** |
| **crea** | **Creatinine** | **mmol/l** |
| **alb** | **Albumin** | **signal area** |
| **gp** | **Glycoprotein acetyls, mainly a1-acid glycoprotein** | **mmol/l** |
| **bOHBut** | **3-hydroxybutyrate** | **mmol/l** |
| **XXL_VLDL_L** | **Total lipids in chylomicrons and extremely large VLDL** | **mmol/l** |
| **XL_VLDL_L** | **Total lipids in very large VLDL** | **mmol/l** |
| **L_VLDL_L** | **Total lipids in large VLDL** | **mmol/l** |
| **XL_HDL_L** | **Total lipids in very large HDL** | **mmol/l** |
| **L_HDL_L** | **Total lipids in large HDL** | **mmol/l** |

**In grey: 6 metabolomic variables measured at low success rates (< 98%) or that frequently failed to reach the detection limit (< 99%) were excluded.**

# Supplemental Documents

## Document S1: Estimating the metabolic surrogates

- 1. **Method selection**

As we mention in the main text, each clinical variable is predicted by a penalized logistic regression model (like Equation 1 in the main text):

| $\pi_{k}=\Pr\left( c_{k}=1\vert X=m \right)=\frac{1}{1+e^{-(\beta_{0}+ \beta^{T}m)}}$ | (1) |
| --- | --- |

in which $c_{k}$ is one of the *k* clinical variables ($c_{k}=\left\{ 0,1 \right\}$), $m$ the matrix with the values of the 56 measured metabolic features selected after Quality Control, $\beta^{T}$ the vector of regression coefficients, and $\beta_{0}$ the intercept. What we call then “surrogate values” are the values $\pi_{k}$, for each observation i, which posterior probability that indicates the likelihood of a sample belonging to one of two labels of the $c_{k}\left\{ 0,1 \right\}$. The models are trained using the R package glmnet^1^. The regression coefficients are found by minimizing the penalized negative binomial log-likelihood:

| $\beta\left( \alpha,\lambda\right)={argmin}_{\beta}( -\left[ \frac{1}{N}\sum_{i}^{N} c_{k,i} \left( \beta_{0}+m_{i}^{T}\beta\right)-log(1+e^{{(\beta}_{0}+m_{i}^{T}\beta)} \right]+\lambda[\alpha\left\Vert\beta\right\Vert_{1}+\frac{1}{2}(1-\alpha)\left\Vert\beta\right\Vert_{2}^{2}])$ | (2) |
| --- | --- |

in which, an L1 ($\left\| \beta\right\|_{1})$ and L2 norm ($\left\| \beta\right\|_{2}^{2})$ regularizations are added to the negative binomial log-likelihood estimation (within square brackets), to avoid overfitting. The regularization is then coordinated by the two hyperparameters: $\lambda$ ($\in(0,\infty)$) which represents the “shrinkage parameter” and $\alpha$ ($\in(0,1)$) which is the mixing parameter balancing the L1 and L2 norm regularizations. It is possible to tune both $\lambda$ and $\alpha$ using cross-validations, however, in our first investigations we observed a great fluctuation in the hyperparameters selection when tuning them both, without a real accuracy improvement (more details in Document S2). Therefore, considering the advantages of the ElasticNET over the other methods, which combine both shrinking and variables selection and we decided to fix the mixing parameter $\alpha$ at 0.5 for the predictive models, like previously done by other authors^2–4^

- 1. **Training and validation procedure**

We employed two training-evaluation procedures to get an unbiased estimate of the models’ possible performances (Figure 1). As a first scenario, we used a Double 5-Fold-Cross-Validation (5-Fold CV) with 5 repetitions. This procedure consists of two loops of 5FCV, one internal and one external, in which we first split the dataset in testing (20%) and training (80%) sets and then on the latter set we have another 5FCV repeated for 5 different times, which is done for an unbiased tuning of the model (setting the correct $\lambda$ parameter) that is finally trained on the complete training dataset and tested on the left-out test data. Both 5-FoldCVs were done such that the original distribution of each clinical variable is maintained as much as possible (using the function *createFolds* from the R package *caret*^5^). In the second training-testing procedure, we applied a Leave-One-Biobank-Out-Validation (LOBOV), which consists of holding out one of the biobanks with the considered variable available, which is then used as a test set, while training on the remaining biobanks.^6^ Also, in this setting, we applied a 5FCV with 5 repetitions to tune the best model for each training set.

## Document S2: On fixing the ElasticNET mixing parameter

In this Supplementary document we show our exploratory analysis, to investigate which of the most popular penalized regression methods (Ridge (RR), Lasso (LR) and ElasticNET Regression (EN), implemented in the R package glmnet^1^, could deliver the most consistent metabolic prediction models for the clinical variables available.

As explained in the Document S1, when doing a penalized logistic regression, the regularization term in the formula can be optimized using two hyperparameters: lambda ($\lambda$), the so called “shrinkage parameter”, and alpha ($\alpha$). The larger the lambda, the greater would be the shrinkage in the regression coefficients. While alpha, the mixing parameter, is used to decide how much of the L1 norm and how much of the L2 norm (ridge regression, alpha=0) it is used in the final model. For alpha=1 we have a lasso regression, for alpha=0 we have a Ridge Regression, while an ElasticNET is obtained with an alpha between 0 and 1.

We therefore performed a 5-Fold Cross Validations (using the function *createFolds* from the R package *caret*^5^*)* to optimize both the hyperparameters within the BBMRI dataset, after Quality control (described in Document S3). What we immediately observed was a great fluctuation of the hyperparameters selection whenever tuning both lambda and alpha.

The following table represent the values of alpha and lambda which were observed to have the best accuracy in predicting sex within each of the 5 Folds.

Table 1: Hyperparameters selection while variating both alpha and lambda for the Sex model

| **Fold** | **Alpha** | **Lambda** | **Accuracy** | **Misclassification** |
| --- | --- | --- | --- | --- |
| **Fold1** | 0.8 | 0.000001 | 0.8369165 | 0.1630835 |
| **Fold2** | 0.5 | 0.000001 | 0.8547637 | 0.1452363 |
| **Fold3** | 0.2 | 0.002 | 0.8442766 | 0.1557234 |
| **Fold4** | 0.3 | 0.001 | 0.8436652 | 0.1563348 |
| **Fold5** | 0.0 | 0.001 | 0.8435018 | 0.1564982 |

While, in the first Fold the best accuracy was obtained with an alpha=0.8, for the 5th Fold the best value of mixing parameter is equal to 0. In this sense, we found counter intuitive to see that in some sets the most accurate model is very close to be a Lasso Regression while other times it is a Ridge regression. Instead, if we fix the mixing parameter to alpha=0.5, the results table is:

Table 2: Hyperparameters selection while variating only lambda for the Sex model

| **Fold** | **Alpha** | **Lambda** | **Accuracy** | **Misclassification** |
| --- | --- | --- | --- | --- |
| **Fold1** | 0.5 | 0.000001 | 0.8367228 | 0.1632772 |
| **Fold2** | 0.5 | 0.00002 | 0.8547637 | 0.1452363 |
| **Fold3** | 0.5 | 0.001 | 0.8435018 | 0.1564982 |
| **Fold4** | 0.5 | 0.00001 | 0.8432778 | 0.1567222 |
| **Fold5** | 0.5 | 0.00003 | 0.8425334 | 0.1574666 |

We can immediately notice that we obtain much more stable selection of the tuning parameters within the 5 Folds. These results were repeated also in the models built to predict the other clinical variables.

Table 3: Hyperparameters selection while variating both alpha and lambda for the Diabetes model

| **Fold** | **Alpha** | **Lambda** | **Accuracy** | **Misclassification** |
| --- | --- | --- | --- | --- |
| **Fold1** | 0.1 | 0.000001 | 0.9192175 | 0.0807825 |
| **Fold2** | 0.7 | 0.000001 | 0.9125432 | 0.0874568 |
| **Fold3** | 0.5 | 0.000003 | 0.9176254 | 0.0823746 |
| **Fold4** | 0.2 | 0.000001 | 0.9162255 | 0.0837745 |
| **Fold5** | 0.0 | 0.000001 | 0.9054085 | 0.0945915 |

Table 4: Hyperparameters selection while variating both alpha and lambda for the Metabolic Syndrome model

| **Fold** | **Alpha** | **Lambda** | **Accuracy** | **Misclassification** |
| --- | --- | --- | --- | --- |
| **Fold1** | 1.0 | 0.005 | 0.8401535 | 0.1598465 |
| **Fold2** | 0.0 | 0.000001 | 0.8650032 | 0.1349968 |
| **Fold3** | 0.7 | 0.000001 | 0.8566859 | 0.1433141 |
| **Fold4** | 0.0 | 0.003 | 0.8523018 | 0.1476982 |
| **Fold5** | 0.0 | 0.000002 | 0.8478261 | 0.1521739 |

Similar results were obtained for all the other binary clinical variables. Considering the fact that the Lasso Regression (alpha=1) is known to be affected by high correlation between variables; while Ridge regression (alpha=1), cannot perform feature selection, we decided to fix the alpha parameter to 0.5.

## Document S3: Discussion upon the use of “*high age*” for the Stepwise Cox regression models.

We decided to split on sex as previous literature has already shown that men and women can be quite different at older ages.^7,8^ However, we were indeed also surprised that the variables selected were extremely different. In particular, it could be logical that ‘*high age*’ would appear to be significant in both males and females, even more, as it is being selected in a model across both genders together too (Figure S8A).

However, we think that there might be a few considerations that could explain this different behavior of the two models. First, it is important to notice that the real age of the subjects was selected as a variable in both models. Therefore, probably most of the variance explained by ‘*s high age’* is already incorporated by the variable ‘age’. Second, ‘*high age*’ was trained to recognize people over 65 years old, while the subject in LLS_SIBS are nonagenarians. Therefore, even though we think that a metabolic indication of high age could be indicative for Time to Death, we think that such a variable could be less informative for such an older population. Indeed, in Figure 2, we show that ‘*high age’* has a moderate correlation with their Age at Death, but that this association is stronger in men (R= -0.16 Figure 3B) than women (R=-0.095, Figure 3C). Third, we speculate that, since the metabolic profiling contains mostly lipids, our surrogate models might capture different aspects of cardiovascular mortality, which is known to be more common in men than women, even at older ages.^9^.

| A)  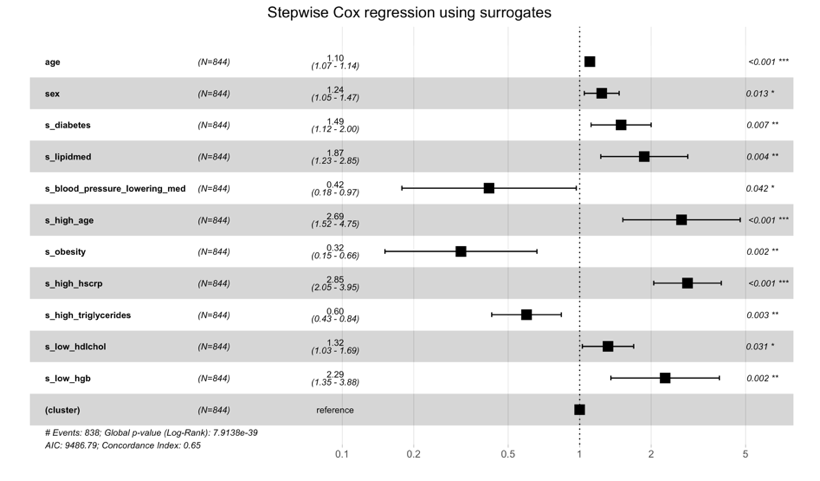 | |
| --- | --- |
| B)  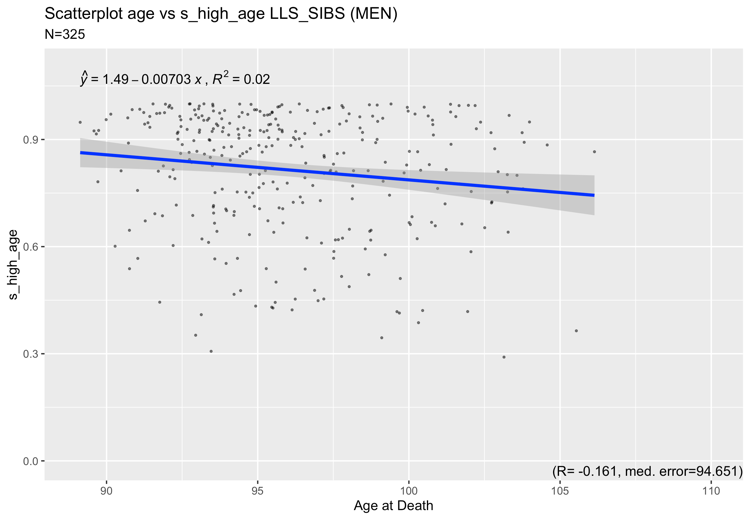 | C)  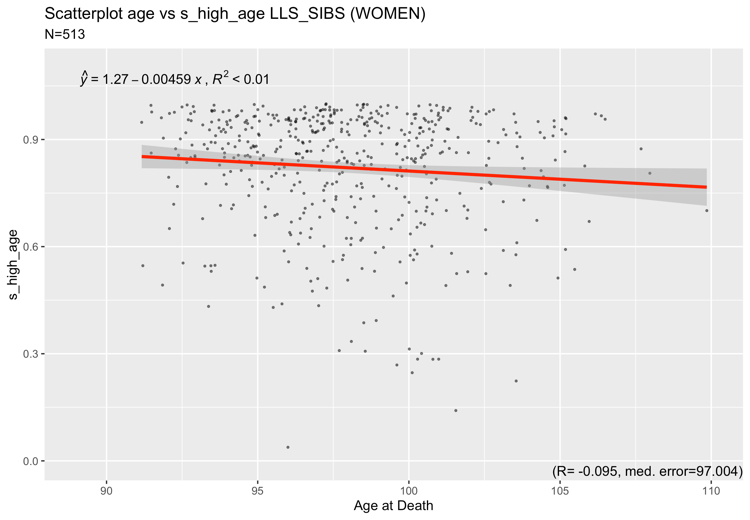 |
| **Figure S8: Associations of high_age with mortality:** A) Stepwise cox regression done on LLS_SIBS, for both men and women together. B) and C) scatterplots of ‘Age at Death’ vs ‘s_high_age’ for men and women, respectively. Note, for illustrative purposes, figures B) and C) have been created using data on subjects who had died during follow-up, which constituted almost the totality of the dataset (N_men_=325 out of 326, N_women_=513 out of 518). | |

# References

1. Friedman J, Hastie T, Tibshirani R. Regularization Paths for Generalized Linear Models via Coordinate Descent. J Stat Softw 2010;33(1):1–22.

2. Lu AT, Quach A, Wilson JG, et al. DNA methylation GrimAge strongly predicts lifespan and healthspan. Aging 2019;11(2):303–327.

3. Horvath S. DNA methylation age of human tissues and cell types. Genome Biol 2013;14(10):R115.

4. Gonzales GB, De Saeger S. Elastic net regularized regression for time-series analysis of plasma metabolome stability under sub-optimal freezing condition. Sci Rep 2018;8(1):3659.

5. Kuhn M. Building Predictive Models in R Using the caret Package. J Stat Softw 2008;28:1–26.

6. van den Akker Erik B., Trompet Stella, Barkey Wolf Jurriaan J.H., et al. Metabolic Age Based on the BBMRI-NL 1H-NMR Metabolomics Repository as Biomarker of Age-related Disease. Circ Genomic Precis Med [homepage on the Internet] [cited 2020 Sep 14];0(0). Available from: https://www.ahajournals.org/doi/10.1161/CIRCGEN.119.002610

7. Hägg S, Jylhävä J. Sex differences in biological aging with a focus on human studies. eLife 2021;10:e63425.

8. Márquez EJ, Chung C, Marches R, et al. Sexual-dimorphism in human immune system aging. Nat Commun 2020;11(1):751.

9. Mikkola TS, Gissler M, Merikukka M, Tuomikoski P, Ylikorkala O. Sex Differences in Age-Related Cardiovascular Mortality. PLoS ONE 2013;8(5):e63347.
