# Supplementary material for: 1H-NMR metabolomics-based surrogates to impute common clinical risk factors and endpoints
Source: eBioMedicine. 2021 Dec 20;75:103764. doi: 10.1016/j.ebiom.2021.103764 (PMC8703237; doi:10.1016/j.ebiom.2021.103764)
Supplement: Supplementary file 2 [file mmc2.docx]

##

Quality Control

The current document contains detailed information regarding the Quality Control performed for the paper: "^1^H-NMR metabolomics-based surrogates to impute common clinical risk factors and endpoints”. It contains also a detailed description of the clinical variables after the Quality control.

Table of Contents

[Quality Control Analysis in BBMRI 2](#_Toc87293023)

[Loading the dataset 2](#_Toc87293024)

[Selecting samples and metabolites 3](#_Toc87293025)

[Imputing and transform 7](#_Toc87293026)

[Table describing the final dataset 11](#_Toc87293027)

[Document S3: Clinical Variables available in BBMRI 16](#_Toc87293028)

## Quality Control Analysis in BBMRI

QC analysis of BBMRI-NL

### Loading the dataset

|| Loading the dataset | Removing discontinued metabolites ‘dag’, ‘dag_tg’, ‘falen’, ‘cla’, ‘cla_fa’ … Done! | Removing metabolites not reported in serum ‘pyr’ … Done! | Removing dependent metabolites ‘run_id’, ‘u2_27ppm’, ‘abnormal_macromolecule_a’, ‘aminocaproic_acid’, ‘apob_apoa1’, ‘citrate’, ‘citrate_plasma’, ‘dha_fa’, ‘diluted’, ‘estc’, ‘ethanol’, ‘fast’, ‘freec’, ‘gln_to_glu’, ‘gluconolactone’, ‘glycerol’, ‘glycine’, ‘hdl_tg’, ‘high_lactate’, ‘high_pyruvate’, ‘idl_c_percentage’, ‘idl_ce’, ‘idl_ce_percentage’, ‘idl_fc’, ‘idl_fc_percentage’, ‘idl_p’, ‘idl_pl’, ‘idl_pl_percentage’, ‘idl_tg’, ‘idl_tg_percentage’, ‘inferred_sample_matrix’, ‘isopropanol’, ‘l_hdl_c’, ‘l_hdl_c_percentage’, ‘l_hdl_ce’, ‘l_hdl_ce_percentage’, ‘l_hdl_fc’, ‘l_hdl_fc_percentage’, ‘l_hdl_p’, ‘l_hdl_pl’, ‘l_hdl_pl_percentage’, ‘l_hdl_tg’, ‘l_hdl_tg_percentage’, ‘l_ldl_c’, ‘l_ldl_c_percentage’, ‘l_ldl_ce’, ‘l_ldl_ce_percentage’, ‘l_ldl_fc’, ‘l_ldl_fc_percentage’, ‘l_ldl_p’, ‘l_ldl_pl’, ‘l_ldl_pl_percentage’, ‘l_ldl_tg’, ‘l_ldl_tg_percentage’, ‘l_vldl_c’, ‘l_vldl_c_percentage’, ‘l_vldl_ce’, ‘l_vldl_ce_percentage’, ‘l_vldl_fc’, ‘l_vldl_fc_percentage’, ‘l_vldl_p’, ‘l_vldl_pl’, ‘l_vldl_pl_percentage’, ‘l_vldl_tg’, ‘l_vldl_tg_percentage’, ‘la_fa’, ‘ldl_tg’, ‘low_glucose’, ‘m_hdl_c’, ‘m_hdl_c_percentage’, ‘m_hdl_ce’, ‘m_hdl_ce_percentage’, ‘m_hdl_fc’, ‘m_hdl_fc_percentage’, ‘m_hdl_p’, ‘m_hdl_pl’, ‘m_hdl_pl_percentage’, ‘m_hdl_tg’, ‘m_hdl_tg_percentage’, ‘m_ldl_c’, ‘m_ldl_c_percentage’, ‘m_ldl_ce’, ‘m_ldl_ce_percentage’, ‘m_ldl_fc’, ‘m_ldl_fc_percentage’, ‘m_ldl_p’, ‘m_ldl_pl’, ‘m_ldl_pl_percentage’, ‘m_ldl_tg’, ‘m_ldl_tg_percentage’, ‘m_vldl_c’, ‘m_vldl_c_percentage’, ‘m_vldl_ce’, ‘m_vldl_ce_percentage’, ‘m_vldl_fc’, ‘m_vldl_fc_percentage’, ‘m_vldl_p’, ‘m_vldl_pl’, ‘m_vldl_pl_percentage’, ‘m_vldl_tg’, ‘m_vldl_tg_percentage’, ‘measurement_date’, ‘plasma’, ‘platform_version’, ‘polysaccharide’, ‘remnant_c’, ‘s_hdl_c’, ‘s_hdl_c_percentage’, ‘s_hdl_ce’, ‘s_hdl_ce_percentage’, ‘s_hdl_fc’, ‘s_hdl_fc_percentage’, ‘s_hdl_p’, ‘s_hdl_pl’, ‘s_hdl_pl_percentage’, ‘s_hdl_tg’, ‘s_hdl_tg_percentage’, ‘s_ldl_c’, ‘s_ldl_c_percentage’, ‘s_ldl_ce’, ‘s_ldl_ce_percentage’, ‘s_ldl_fc’, ‘s_ldl_fc_percentage’, ‘s_ldl_p’, ‘s_ldl_pl’, ‘s_ldl_pl_percentage’, ‘s_ldl_tg’, ‘s_ldl_tg_percentage’, ‘s_vldl_c’, ‘s_vldl_c_percentage’, ‘s_vldl_ce’, ‘s_vldl_ce_percentage’, ‘s_vldl_fc’, ‘s_vldl_fc_percentage’, ‘s_vldl_p’, ‘s_vldl_pl’, ‘s_vldl_pl_percentage’, ‘s_vldl_tg’, ‘s_vldl_tg_percentage’, ‘serum_sample’, ‘singlet_and_triplet_around_2_5’, ‘solvent_peak_ch3’, ‘tg_pg’, ‘unexpected_amino_acid_signals’, ‘unknown_acetylated_compound’, ‘unknown_small_molecule_a’, ‘unknown_small_molecule_b’, ‘vldl_tg’, ‘xl_hdl_c’, ‘xl_hdl_c_percentage’, ‘xl_hdl_ce’, ‘xl_hdl_ce_percentage’, ‘xl_hdl_fc’, ‘xl_hdl_fc_percentage’, ‘xl_hdl_p’, ‘xl_hdl_pl’, ‘xl_hdl_pl_percentage’, ‘xl_hdl_tg’, ‘xl_hdl_tg_percentage’, ‘xl_vldl_c’, ‘xl_vldl_c_percentage’, ‘xl_vldl_ce’, ‘xl_vldl_ce_percentage’, ‘xl_vldl_fc’, ‘xl_vldl_fc_percentage’, ‘xl_vldl_p’, ‘xl_vldl_pl’, ‘xl_vldl_pl_percentage’, ‘xl_vldl_tg’, ‘xl_vldl_tg_percentage’, ‘xs_vldl_c’, ‘xs_vldl_c_percentage’, ‘xs_vldl_ce’, ‘xs_vldl_ce_percentage’, ‘xs_vldl_fc’, ‘xs_vldl_fc_percentage’, ‘xs_vldl_p’, ‘xs_vldl_pl’, ‘xs_vldl_pl_percentage’, ‘xs_vldl_tg’, ‘xs_vldl_tg_percentage’, ‘xxl_vldl_c’, ‘xxl_vldl_c_percentage’, ‘xxl_vldl_ce’, ‘xxl_vldl_ce_percentage’, ‘xxl_vldl_fc’, ‘xxl_vldl_fc_percentage’, ‘xxl_vldl_p’, ‘xxl_vldl_pl’, ‘xxl_vldl_pl_percentage’, ‘xxl_vldl_tg’, ‘xxl_vldl_tg_percentage’, ‘visit_id’ … Done!

Removing individuals younger than 18 y. o. 4

Calculating BMI, LDL-cholesterol and eGFR using CKD-EPI

|  | metabolites | phenotypes |
| --- | --- | --- |
| Rows | 31,322 | 31,322 |
| Columns | 62 | 79 |

### Selecting samples and metabolites

Missing values per biobank

|Excluding 4531 samples, 4385 entries from VUNTR and 146 entries from CODAM | Removing metabolites showing many missing or zero values ‘bohbut’ … Done!

Zeros per biobank

Removing metabolites showing many missing or zero values ‘xl_vldl_l’, ‘xxl_vldl_l’, ‘l_vldl_l’, ‘xl_hdl_l’, ‘l_hdl_l’ … Done!

Missing values per sample

Removing 270 entries with missing values [Nmax>1] … ALPHAOMEGA [N=5]; BIOMARCS [N=58]; CHECK [N=5]; CSF [N=2]; DMS [N=3]; DZS_WF [N=7]; ERF [N=7]; FUNCTGENOMICS [N=8]; GARP [N=1]; HELIUS [N=30]; HOF [N=4]; LIFELINES [N=7]; LLS_PARTOFFS [N=2]; LLS_SIBS [N=4]; NESDA [N=33]; PROSPER [N=6]; RAAK [N=3]; RS [N=14]; STEMI_GIPS-III [N=1]; TACTICS [N=17]; TOMAAT [N=4]; UCORBIO [N=37]; VUMC_ADC [N=12] … Done!

Zeros per sample

Removing 70 entries with zero values [Nmax>1] … BIOMARCS [N=8]; CHECK [N=1]; CSF [N=1]; DMS [N=10]; DZS_WF [N=7]; FUNCTGENOMICS [N=2]; HELIUS [N=12]; LIFELINES [N=7]; LLS_PARTOFFS [N=3]; NESDA [N=4]; RS [N=5]; STEMI_GIPS-III [N=3]; UCORBIO [N=4]; VUMC_ADC [N=3] … Done!

5 Standard Deviation

Removing 584 entries with a 5SD outlier … ALPHAOMEGA [N=101]; BIOMARCS [N=32]; CHARM [N=2]; CHECK [N=4]; CSF [N=2]; DMS [N=27]; DZS_WF [N=35]; ERF [N=5]; FUNCTGENOMICS [N=30]; GARP [N=10]; HELIUS [N=15]; HOF [N=9]; LIFELINES [N=17]; LLS_PARTOFFS [N=52]; LLS_SIBS [N=28]; MRS [N=1]; NESDA [N=33]; PROSPER [N=29]; RAAK [N=4]; RS [N=73]; STABILITEIT [N=1]; STEMI_GIPS-III [N=15]; TACTICS [N=2]; TOMAAT [N=5]; UCORBIO [N=40]; VUMC_ADC [N=12] … Done!

### Imputing and transform

Barplot of the metabolites before transformation

| 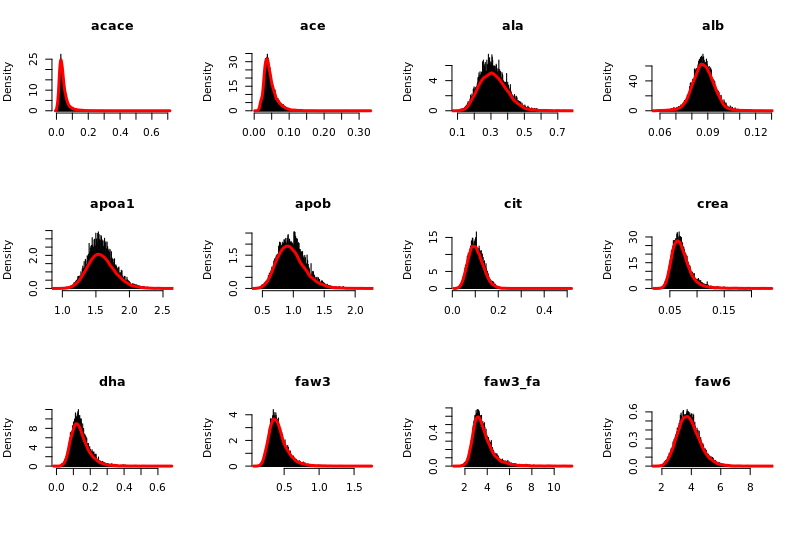 | 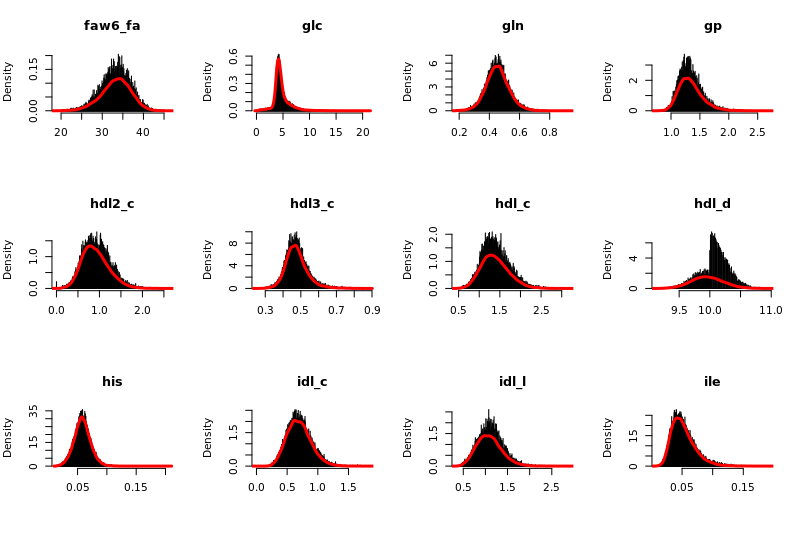 |
| --- | --- |
| 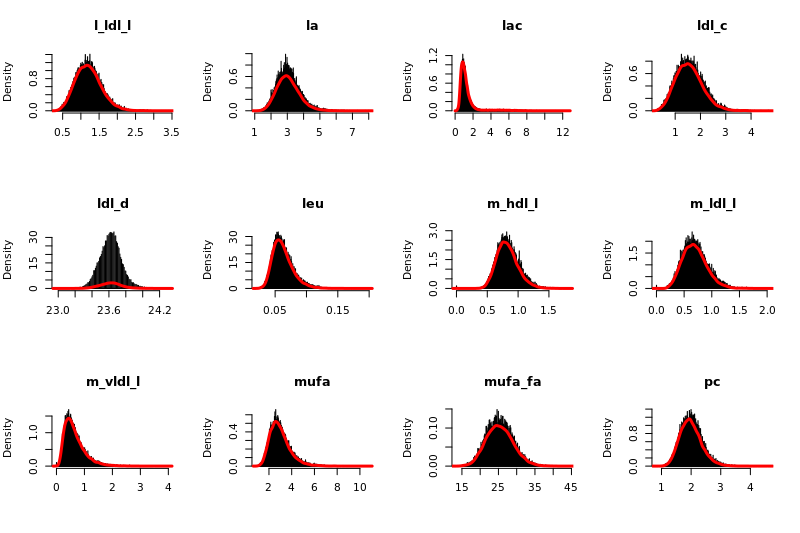 | 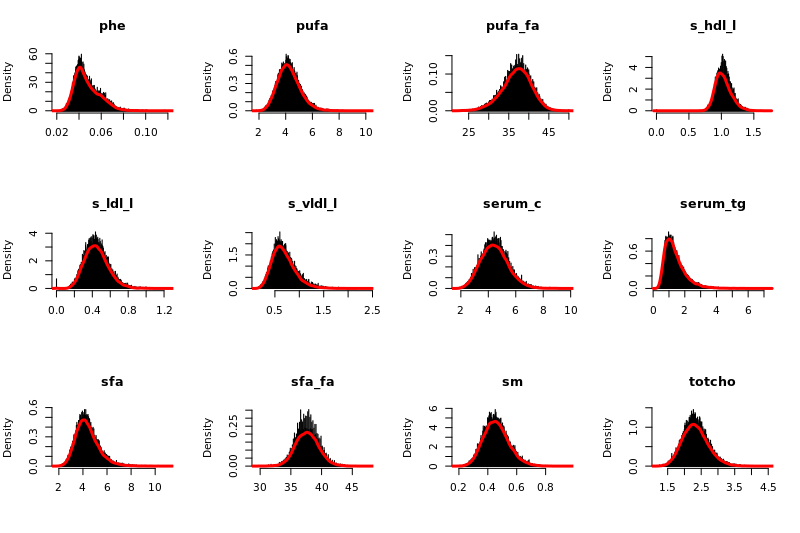 |


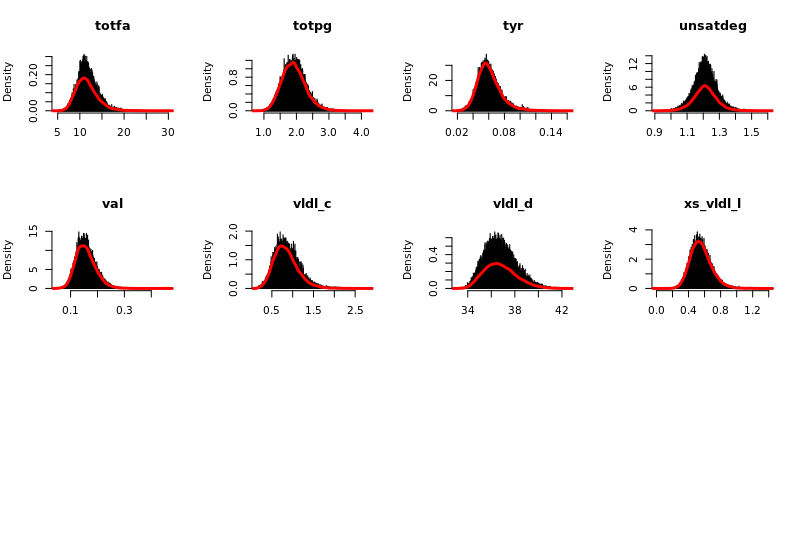


Imputing and transforming

Imputing 534 [ 0.037 %] missing values …


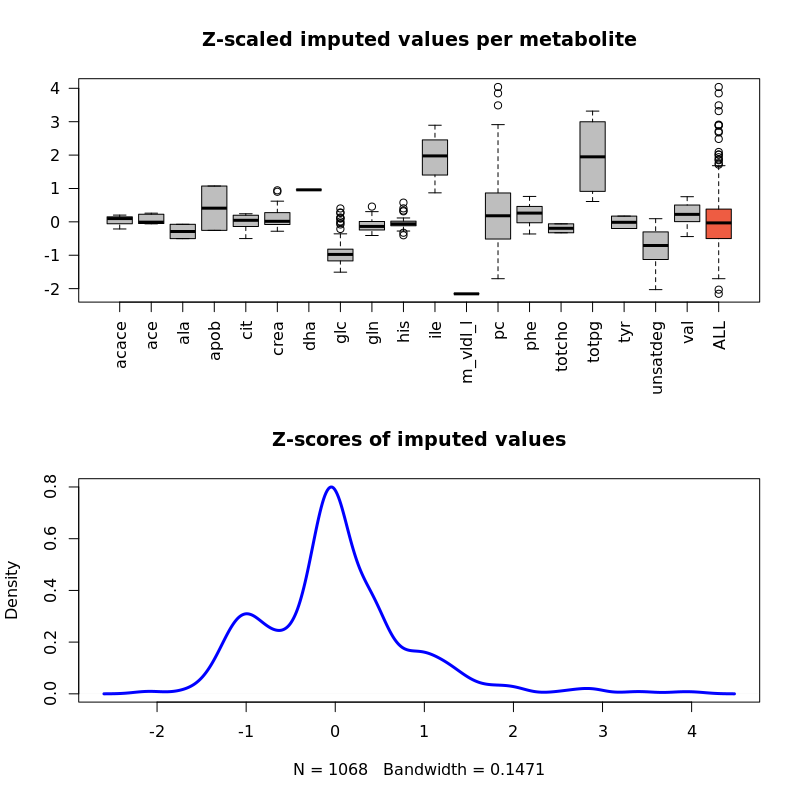


Done!
Apply scaling … Done!


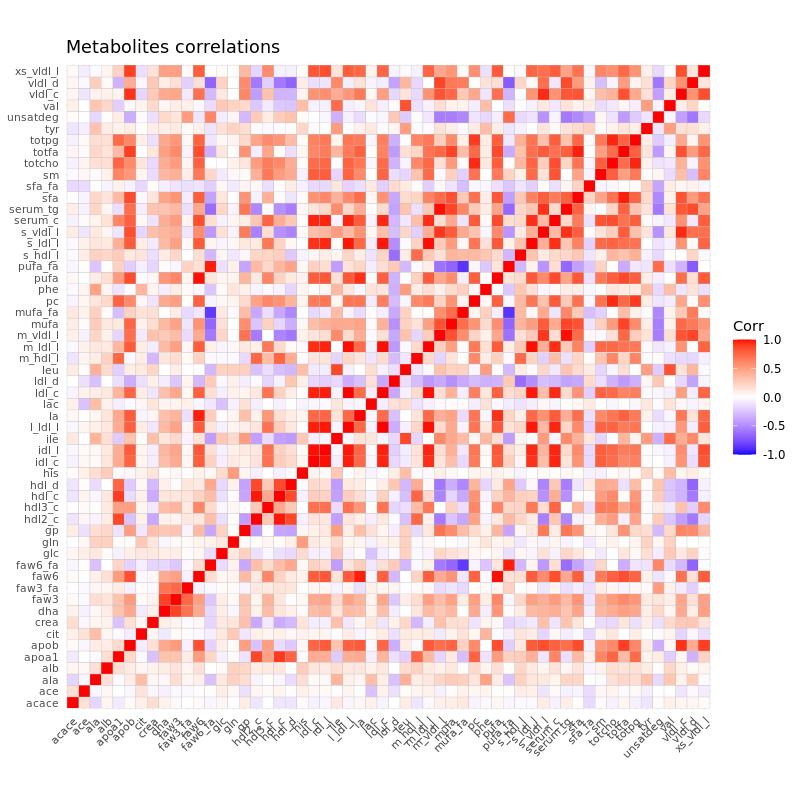


|  | metabolites | phenotypes |
| --- | --- | --- |
| Rows | 25.867 | 25.867 |
| Columns | 56 | 79 |

Bar plot of the metabolites after imputation and z-transformation

| 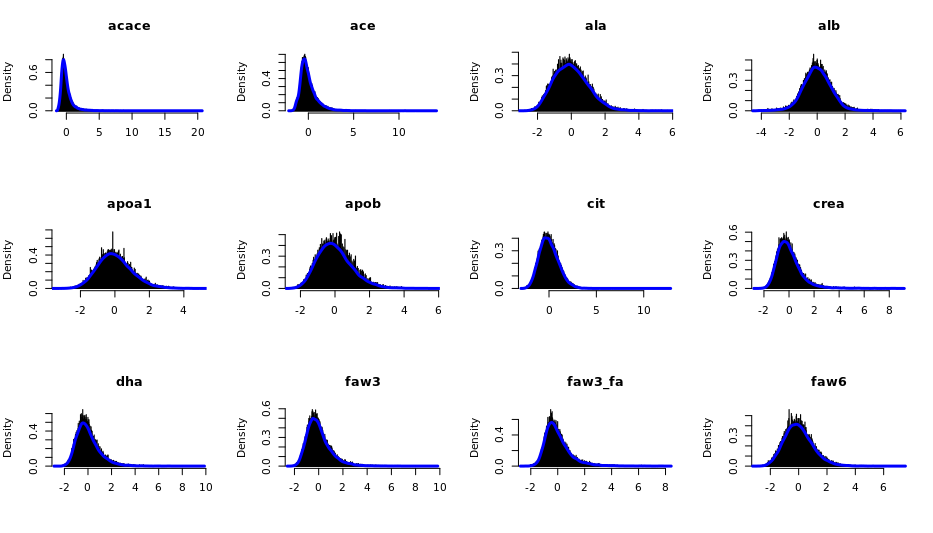 | 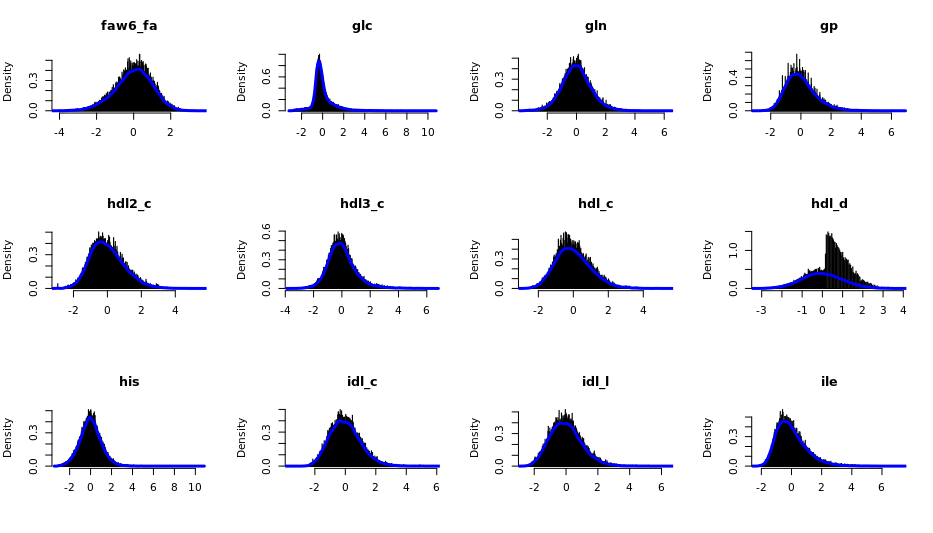 |
| --- | --- |
| 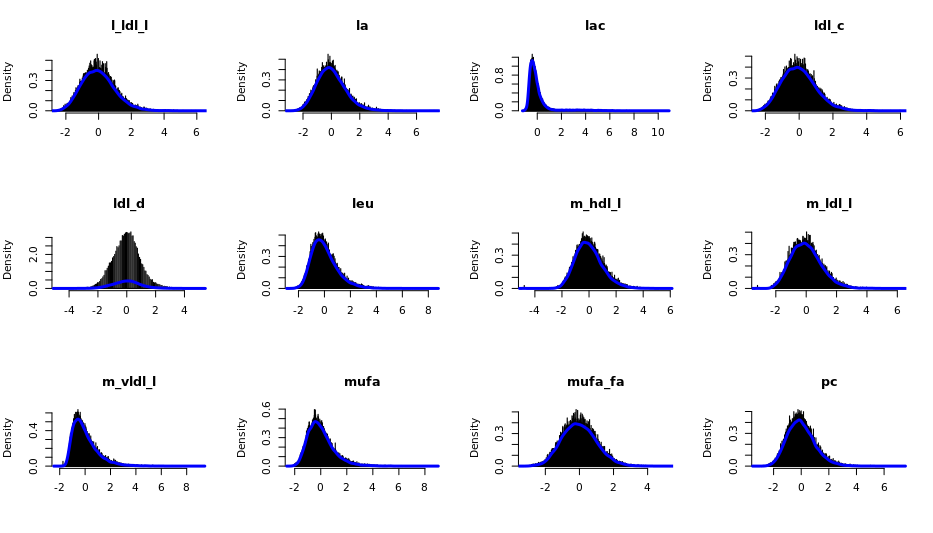 | 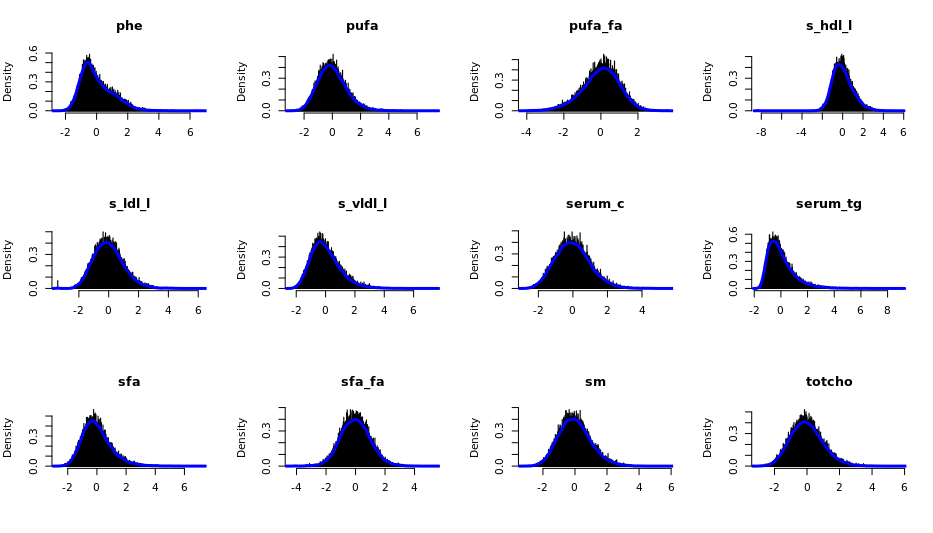 |


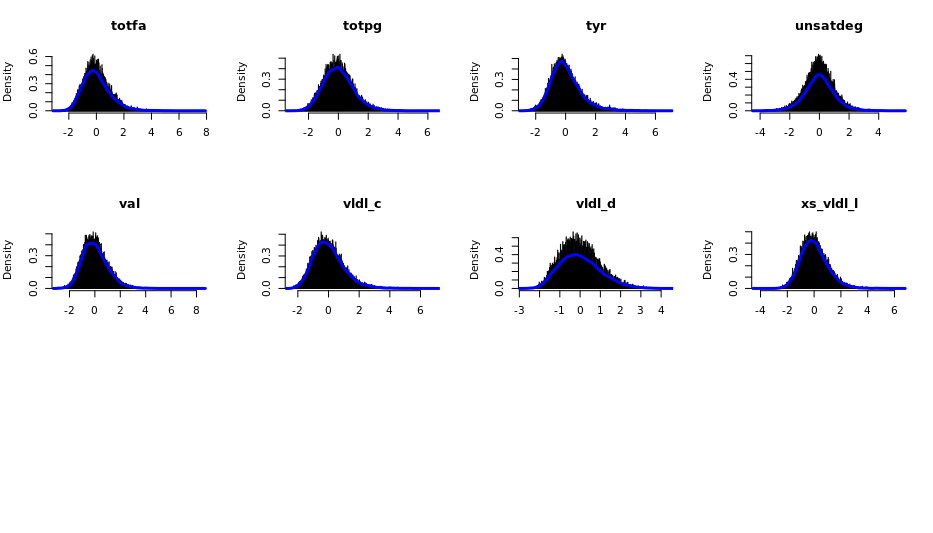


### Table describing the final dataset

|  | N | Males % | Missing Age | Age | Missing BMI | BMI | Missing diabetes | TRUE diabetes | FALSE diabetes |
| --- | --- | --- | --- | --- | --- | --- | --- | --- | --- |
| **LIFELINES** | 1456 | 42.0 | 1 | 45.0 [18-85] | 1 | 25.3 [15.81-44.92] | 43 | 31 | 1382 |
| **LLS_PARTOFFS** | 2291 | 44.4 | 0 | 58.8 [30-82] | 336 | 25.4 [16.33-46.78] | 354 | 95 | 1842 |
| **RS** | 5063 | 42.0 | 52 | 70.1 [37-98] | 131 | 27.4 [14.15-53.26] | 351 | 554 | 4158 |
| **NESDA** | 2381 | 33.4 | 0 | 44.2 [18-72] | 40 | 25.9 [15.84-55.83] | 0 | 102 | 2279 |
| **VUMC_ADC** | 1446 | 55.5 | 0 | 64.0 [27-88] | 291 | 25.3 [14.82-43.76] | 351 | 76 | 1019 |
| **CHARM** | 124 | 24.2 | 0 | 46.3 [19-65] | 31 | 24.7 [18.5-48.98] | 124 | 0 | 0 |
| **TACTICS** | 79 | 27.8 | 3 | 71.5 [45-88] | 79 | NaN | 79 | 0 | 0 |
| **PROSPER** | 948 | 51.2 | 0 | 75.6 [70-83] | 0 | 27.1 [15.61-43.86] | 0 | 122 | 826 |
| **FUNCTGENOMICS** | 460 | 63.7 | 3 | 42.2 [18-98] | 245 | 22.6 [15.09-35.43] | 236 | 1 | 223 |
| **STEMI_GIPS-III** | 307 | 76.5 | 0 | 58.7 [23-88] | 11 | 26.8 [18.62-46.87] | 0 | 0 | 307 |
| **GARP** | 424 | 20.3 | 32 | 59.2 [30-79] | 39 | 27.1 [19.1-46.85] | 424 | 0 | 0 |
| **UCORBIO** | 1117 | 72.3 | 0 | 64.5 [18-93] | 82 | 27.1 [15.47-50.03] | 3 | 236 | 878 |
| **DMS** | 1813 | 57.8 | 0 | 60.8 [40-80] | 2 | 27.3 [17.18-52.25] | 0 | 947 | 866 |
| **CSF** | 254 | 39.8 | 0 | 41.1 [18-69] | 2 | 24.0 [17.72-33.69] | 0 | 1 | 253 |
| **LLS_SIBS** | 966 | 37.6 | 0 | 92.0 [53-103] | 966 | NaN | 966 | 0 | 0 |
| **RAAK** | 82 | 32.9 | 3 | 68.1 [25-85] | 3 | 28.8 [18.4-37.95] | 82 | 0 | 0 |
| **DZS_WF** | 1151 | 56.0 | 0 | 63.1 [33-89] | 1 | 30.4 [19.71-57.1] | 0 | 1151 | 0 |
| **ERF** | 1389 | 44.7 | 3 | 47.7 [18-86] | 5 | 26.9 [15.54-51.13] | 20 | 33 | 1336 |
| **TOMAAT** | 225 | 38.7 | 1 | 63.6 [18-91] | 225 | NaN | 0 | 20 | 205 |
| **HELIUS** | 443 | 40.0 | 0 | 52.2 [19-69] | 1 | 30.5 [19.29-53.6] | 0 | 443 | 0 |
| **CHECK** | 969 | 20.6 | 4 | 56.0 [44-66] | 969 | NaN | 969 | 0 | 0 |
| **MRS** | 89 | 50.6 | 0 | 35.1 [19-52] | 0 | 23.1 [18.51-28.46] | 0 | 0 | 89 |
| **BIOMARCS** | 532 | 78.2 | 2 | 61.0 [32-87] | 3 | 27.6 [16.79-58.76] | 3 | 135 | 394 |
| **ALPHAOMEGA** | 771 | 76.9 | 0 | 69.6 [59-80] | 1 | 27.6 [17.75-47.34] | 0 | 190 | 581 |
| **STABILITEIT** | 136 | 48.5 | 0 | 67.7 [64-72] | 136 | NaN | 136 | 0 | 0 |
| **HOF** | 951 | 46.9 | 39 | 58.2 [36-77] | 42 | 27.5 [17.35-50.62] | 0 | 114 | 837 |

|  | Not using lipidmed | Using statins | Using other lipidmed | Missing  current  smoking | TRUE  Current  smoking | FALSE  Current  smoking | Missing  Diastolic Pressure | Diastolic Pressure | Missing Systolic  Presssure | Systolic  Pressure |
| --- | --- | --- | --- | --- | --- | --- | --- | --- | --- | --- |
| **LIFELINES** | 1281 | 58 | 1 | 0 | 294 | 1162 | 36 | 70.9 [50-104] | 36 | 120.0 [87-194] |
| **LLS_PARTOFFS** | 1868 | 201 | 5 | 0 | 875 | 1416 | 2257 | 85.7 [69-106.25] | 2257 | 148.5 [114.75-212.75] |
| **RS** | 3859 | 1158 | 0 | 0 | 1997 | 3066 | 776 | 80.5 [39-128] | 776 | 145.4 [75-240] |
| **NESDA** | 2186 | 185 | 10 | 0 | 900 | 1481 | 43 | 80.0 [50-120.5] | 43 | 133.7 [90.5-221.5] |
| **VUMC_ADC** | 729 | 204 | 0 | 0 | 338 | 1108 | 174 | 85.9 [34-125] | 173 | 141.2 [64-220] |
| **CHARM** | 0 | 0 | 0 | 0 | 59 | 65 | 124 | NaN | 124 | NaN |
| **TACTICS** | 0 | 0 | 0 | 0 | 79 | 0 | 79 | NaN | 79 | NaN |
| **PROSPER** | 948 | 0 | 0 | 0 | 254 | 694 | 0 | 82.9 [50-130] | 0 | 154.7 [96-225] |
| **FUNCTGENOMICS** | 0 | 0 | 0 | 0 | 264 | 196 | 460 | NaN | 460 | NaN |
| **STEMI_GIPS-III** | 4 | 303 | 0 | 0 | 162 | 145 | 2 | 73.2 [50-120] | 2 | 123.9 [80-205] |
| **GARP** | 0 | 0 | 0 | 0 | 424 | 0 | 424 | NaN | 424 | NaN |
| **UCORBIO** | 465 | 639 | 9 | 0 | 636 | 481 | 119 | 72.0 [37.5-131.67] | 120 | 131.6 [82.43-211.5] |
| **DMS** | 969 | 827 | 15 | 0 | 246 | 1567 | 1 | 75.6 [47.33-108.67] | 1 | 136.6 [88-214.5] |
| **CSF** | 247 | 7 | 0 | 0 | 43 | 211 | 254 | NaN | 254 | NaN |
| **LLS_SIBS** | 0 | 0 | 0 | 0 | 966 | 0 | 966 | NaN | 966 | NaN |
| **RAAK** | 0 | 0 | 0 | 0 | 82 | 0 | 82 | NaN | 82 | NaN |
| **DZS_WF** | 296 | 855 | 0 | 0 | 237 | 914 | 6 | 76.3 [41-117] | 6 | 142.7 [95-243] |
| **ERF** | 1226 | 155 | 0 | 0 | 541 | 848 | 110 | 82.5 [58.5-113] | 110 | 131.8 [92-179.5] |
| **TOMAAT** | 0 | 0 | 0 | 0 | 225 | 0 | 225 | NaN | 225 | NaN |
| **HELIUS** | 326 | 117 | 0 | 0 | 66 | 377 | 1 | 85.5 [62-133] | 0 | 140.7 [100-245] |
| **CHECK** | 0 | 0 | 0 | 0 | 969 | 0 | 969 | NaN | 969 | NaN |
| **MRS** | 89 | 0 | 0 | 0 | 10 | 79 | 89 | NaN | 89 | NaN |
| **BIOMARCS** | 11 | 510 | 4 | 0 | 229 | 303 | 473 | 81.1 [38-118] | 4 | 138.8 [60-220] |
| **ALPHAOMEGA** | 120 | 651 | 0 | 0 | 125 | 646 | 1 | 79.6 [40.5-118.5] | 1 | 141.4 [78.5-219.5] |
| **STABILITEIT** | 0 | 0 | 0 | 0 | 136 | 0 | 136 | NaN | 136 | NaN |
| **HOF** | 116 | 114 | 687 | 0 | 214 | 737 | 39 | 85.9 [58.5-130] | 39 | 140.4 [94-216.5] |

Table continues below

|  | Missing  hsCRP | hsCRP | Missing  Waist Circ | Waist  Circumference | Missing  Metabolic  Syndrome | TRUE Metabolic  Syndrome | FALSE  Metabolic  Syndrome |
| --- | --- | --- | --- | --- | --- | --- | --- |
| **LIFELINES** | 1347 | 2.8 [0-28] | 36 | 88.7 [62-133] | 1456 | 0 | 0 |
| **LLS_PARTOFFS** | 62 | 3.0 [0-220] | 2257 | 99.2 [75.5-132.5] | 2284 | 2 | 5 |
| **RS** | 5063 | NaN | 55 | 94.1 [53.7-176.4] | 5063 | 0 | 0 |
| **NESDA** | 10 | 2.7 [0-75] | 45 | 104.3 [69-175] | 38 | 481 | 1862 |
| **VUMC_ADC** | 1446 | NaN | 795 | 95.9 [62-144] | 1446 | 0 | 0 |
| **CHARM** | 124 | NaN | 124 | NaN | 124 | 0 | 0 |
| **TACTICS** | 79 | NaN | 79 | NaN | 79 | 0 | 0 |
| **PROSPER** | 10 | 6.4 [0-156] | 948 | NaN | 948 | 0 | 0 |
| **FUNCTGENOMICS** | 338 | 12.3 [5-353] | 460 | NaN | 460 | 0 | 0 |
| **STEMI_GIPS-III** | 64 | 4.2 [0-52] | 307 | NaN | 307 | 0 | 0 |
| **GARP** | 424 | NaN | 424 | NaN | 424 | 0 | 0 |
| **UCORBIO** | 137 | 6.2 [0-167] | 259 | 105.7 [39-703] | 1117 | 0 | 0 |
| **DMS** | 1612 | 3.4 [0-53] | 2 | 96.9 [62-152.5] | 4 | 798 | 1011 |
| **CSF** | 254 | NaN | 254 | NaN | 0 | 0 | 254 |
| **LLS_SIBS** | 17 | 6.5 [0-174] | 966 | NaN | 966 | 0 | 0 |
| **RAAK** | 82 | NaN | 82 | NaN | 82 | 0 | 0 |
| **DZS_WF** | 1151 | NaN | 1151 | NaN | 0 | 1151 | 0 |
| **ERF** | 1389 | NaN | 6 | 87.0 [54.3-146.6] | 1389 | 0 | 0 |
| **TOMAAT** | 225 | NaN | 225 | NaN | 225 | 0 | 0 |
| **HELIUS** | 443 | NaN | 1 | 101.0 [72.05-148.9] | 0 | 365 | 78 |
| **CHECK** | 969 | NaN | 969 | NaN | 969 | 0 | 0 |
| **MRS** | 89 | NaN | 89 | NaN | 0 | 0 | 89 |
| **BIOMARCS** | 130 | 12.1 [0-438] | 532 | NaN | 532 | 0 | 0 |
| **ALPHAOMEGA** | 19 | 3.9 [0-79] | 3 | 101.8 [62.5-134] | 0 | 346 | 425 |
| **STABILITEIT** | 136 | NaN | 136 | NaN | 136 | 0 | 0 |
| **HOF** | 951 | NaN | 39 | 97.8 [65.5-140.25] | 0 | 311 | 640 |

Table continues below

|  | Missing alcohol  use | TRUE  Alcohol use | FALSE alcohol use | Missing  Blood pressure lowering  medication | TRUE  Blood pressure lowering  medication | FALSE  Blood pressure lowering  medication | Missing  totchol | totchol |
| --- | --- | --- | --- | --- | --- | --- | --- | --- |
| **LIFELINES** | 1456 | 0 | 0 | 1456 | 0 | 0 | 36 | 5.0 [2.4-9.7] |
| **LLS_PARTOFFS** | 371 | 1720 | 200 | 922 | 261 | 1108 | 55 | 5.6 [1.03-10.84] |
| **RS** | 94 | 4153 | 816 | 742 | 1944 | 2377 | 40 | 5.6 [2.2-12.4] |
| **NESDA** | 49 | 1915 | 417 | 0 | 348 | 2033 | 22 | 5.1 [2.1-9.37] |
| **VUMC_ADC** | 154 | 874 | 418 | 488 | 286 | 672 | 1446 | NaN |
| **CHARM** | 50 | 43 | 31 | 124 | 0 | 0 | 124 | NaN |
| **TACTICS** | 79 | 0 | 0 | 79 | 0 | 0 | 79 | NaN |
| **PROSPER** | 0 | 503 | 445 | 0 | 712 | 236 | 0 | 5.6 [3.85-9.15] |
| **FUNCTGENOMICS** | 237 | 203 | 20 | 460 | 0 | 0 | 460 | NaN |
| **STEMI_GIPS-III** | 307 | 0 | 0 | 0 | 303 | 4 | 36 | 3.8 [2.2-6.6] |
| **GARP** | 424 | 0 | 0 | 424 | 0 | 0 | 424 | NaN |
| **UCORBIO** | 216 | 709 | 192 | 4 | 827 | 286 | 453 | 4.6 [2-9.3] |
| **DMS** | 10 | 1376 | 427 | 3 | 853 | 957 | 1 | 5.0 [2.1-10.4] |
| **CSF** | 0 | 192 | 62 | 0 | 27 | 227 | 254 | NaN |
| **LLS_SIBS** | 966 | 0 | 0 | 966 | 0 | 0 | 966 | NaN |
| **RAAK** | 82 | 0 | 0 | 82 | 0 | 0 | 82 | NaN |
| **DZS_WF** | 200 | 643 | 308 | 0 | 787 | 364 | 6 | 4.6 [1.62-8] |
| **ERF** | 4 | 7 | 1378 | 618 | 249 | 522 | 20 | 5.6 [2.2-10.2] |
| **TOMAAT** | 225 | 0 | 0 | 225 | 0 | 0 | 225 | NaN |
| **HELIUS** | 4 | 239 | 200 | 0 | 220 | 223 | 0 | 4.9 [2.36-8.38] |
| **CHECK** | 969 | 0 | 0 | 969 | 0 | 0 | 969 | NaN |
| **MRS** | 0 | 80 | 9 | 0 | 2 | 87 | 89 | NaN |
| **BIOMARCS** | 532 | 0 | 0 | 20 | 507 | 5 | 119 | 5.0 [2.5-10.2] |
| **ALPHAOMEGA** | 4 | 598 | 169 | 0 | 693 | 78 | 16 | 4.8 [2.59-10.4] |
| **STABILITEIT** | 136 | 0 | 0 | 136 | 0 | 0 | 136 | NaN |
| **HOF** | 0 | 731 | 220 | 0 | 188 | 763 | 39 | 5.7 [1.69-9.98] |

Table continues below

|  | Missing  tryglycerides | tryglicerides | Missing  ldlchol | ldlchol | Missing  hdlchol | hdlchol |
| --- | --- | --- | --- | --- | --- | --- |
| **LIFELINES** | 36 | 1.1 [0.22-14.05] | 44 | 3.0 [0.8435-7.378] | 36 | 1.5 [0.6-3.3] |
| **LLS_PARTOFFS** | 55 | 1.8 [0.12-8.16] | 106 | 3.4 [0.566-7.8785] | 55 | 1.4 [0.16-3.31] |
| **RS** | 2892 | 1.4 [0.34-7.76] | 2902 | 3.4 [0.913-10.225] | 40 | 1.5 [0.41-4.03] |
| **NESDA** | 23 | 1.3 [0.28-7.2] | 41 | 3.0 [0.37-6.81] | 26 | 1.6 [0.57-3.8] |
| **VUMC_ADC** | 1446 | NaN | 1446 | NaN | 1446 | NaN |
| **CHARM** | 124 | NaN | 124 | NaN | 124 | NaN |
| **TACTICS** | 79 | NaN | 79 | NaN | 79 | NaN |
| **PROSPER** | 0 | 1.5 [0.42-4.67] | 4 | 3.7 [1.8425-7.141] | 0 | 1.3 [0.65-3.3] |
| **FUNCTGENOMICS** | 460 | NaN | 460 | NaN | 460 | NaN |
| **STEMI_GIPS-III** | 292 | 1.3 [0.59-3.1] | 292 | 1.9 [1.4555-2.523] | 34 | 1.1 [0.5-2.2] |
| **GARP** | 424 | NaN | 424 | NaN | 424 | NaN |
| **UCORBIO** | 500 | 1.7 [0.2-8] | 515 | 2.6 [0.145-6.445] | 504 | 1.2 [0.3-2.79] |
| **DMS** | 1 | 1.4 [0.29-6.12] | 7 | 2.8 [0.476-7.634] | 1 | 1.5 [0.5-4.6] |
| **CSF** | 254 | NaN | 254 | NaN | 254 | NaN |
| **LLS_SIBS** | 966 | NaN | 966 | NaN | 966 | NaN |
| **RAAK** | 82 | NaN | 82 | NaN | 82 | NaN |
| **DZS_WF** | 6 | 1.8 [0.32-5.85] | 24 | 2.6 [-0.4005-5.678] | 6 | 1.2 [0.41-2.49] |
| **ERF** | 20 | 1.3 [0.1-6.9] | 33 | 3.7 [1.34-7.3] | 20 | 1.3 [0.2-3.1] |
| **TOMAAT** | 225 | NaN | 225 | NaN | 225 | NaN |
| **HELIUS** | 0 | 1.0 [0.27-5.77] | 2 | 3.0 [0.8685-6.1575] | 0 | 1.5 [0.52-3.83] |
| **CHECK** | 969 | NaN | 969 | NaN | 969 | NaN |
| **MRS** | 89 | NaN | 89 | NaN | 89 | NaN |
| **BIOMARCS** | 148 | 1.7 [0.3-11] | 169 | 3.2 [0.143-8.37] | 146 | 1.1 [0.5-4.15] |
| **ALPHAOMEGA** | 16 | 1.9 [0.4641-8.76] | 40 | 2.6 [0.592-8.252] | 16 | 1.3 [0.61-2.69] |
| **STABILITEIT** | 136 | NaN | 136 | NaN | 136 | NaN |
| **HOF** | 39 | 1.5 [0.29-6.24] | 44 | 3.4 [0.83-7.36] | 39 | 1.6 [0.18-4.3] |

Table continues below

## Document S3: Clinical Variables available in BBMRI

In this document we show the availability of each clinical variables that we used in the analyses of: “1H-NMR metabolomics-based surrogates to impute common clinical risk factors and endpoints”. To emphasize the relevant clinical conditions, we used clinical thresholds to obtain dichotomous variables out of the set of the available continuous risk factors, separating between “normal” and “at risk” levels for each risk factor. In this way we obtained: high age, middle age, low age, obesity, high hsCRP, high pressure, high triglycerides, high ldl cholesterol, high total cholesterol. We defined high age, middle age and low age by dividing the ages over all the individuals in BBMRI in tertiles: low age if age < 45 years, middle age if 45 years ≥ age < 65 years and high age for age ≥ 65 years. The variable obesity is a composite variable which separate the individuals based on the following rules: if males and BMI ≥ 30 kg/m^2^ and waist circumference ≥ 102 cm; if females and BMI ≥ 30 kg/m^2^ and waist circumference ≥ 93 cm^7^. We considered 3 mg/L as a threshold for dangerous levels of hsCRP^8^. If systolic blood pressure and diastolic blood pressure were respectively above 140 mmHg and 90 mmHg we considered the individuals with high blood pressure levels^9^. We defined also separate variables for high levels of triglycerides (if triglycerides ≥ 2.3 mmol/L), ldl cholesterol (if ldl cholesterol ≥ 4.1 mmol/L), hdl cholesterol (if hdl cholesterol ≤ 1.03 mmol/L) and total cholesterol (if total cholesterol ≥ 6.2 mmol/L)^9^. People with eGFR≤60 ml/min are considered having moderate loss of kidney function^10^. We considered problematic levels of white blood cell counts if 4.5x10^9^L ≤ white blood cells ≥ 11.0x10^9^L^11^. Finally, dangerous levels of hemoglobin are based on the following rules: if males 2.09 mmol/L ≤ hemoglobin ≥ 2.71 mmol/L; if female 1.86 mmol/L ≤ hemoglobin ≥ 2.48 mmol/L^11^.

#### Binary clinical variables

| 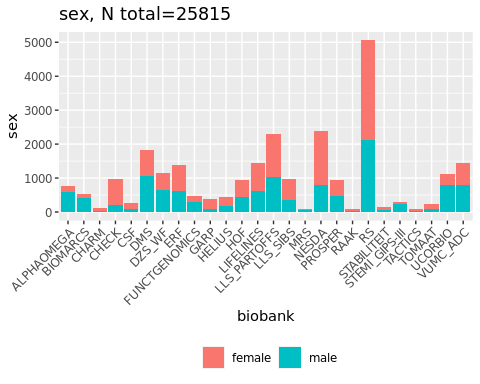 | 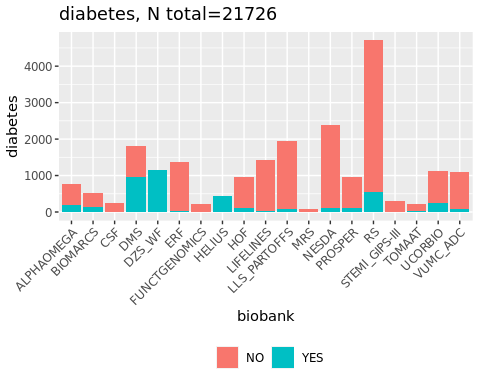 |
| --- | --- |
| 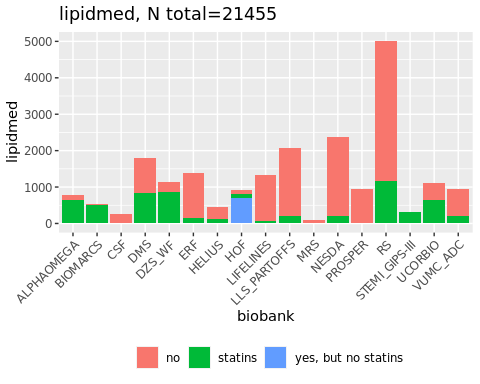 | 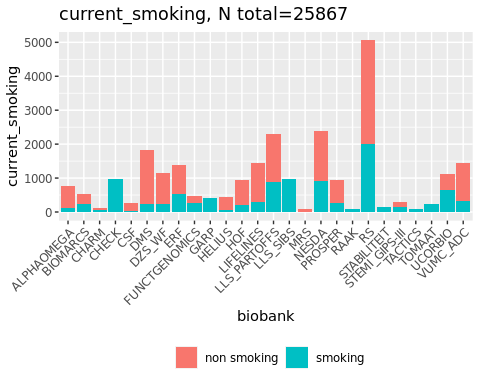 |
| 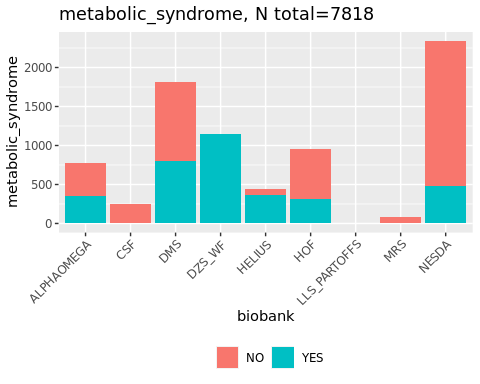 | 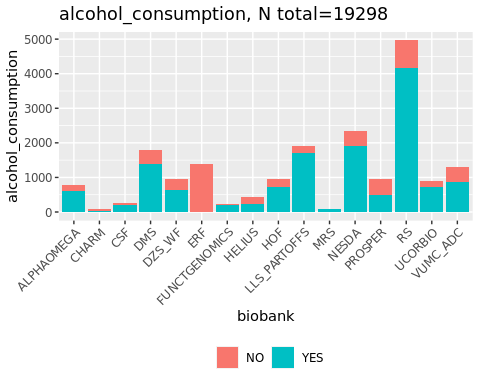 |
| 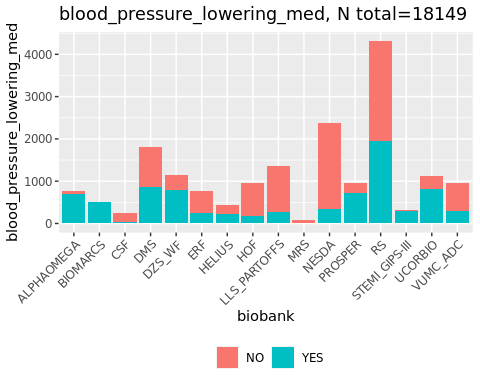 |  |

#### Continuous clinical variables

Age

| Original variable  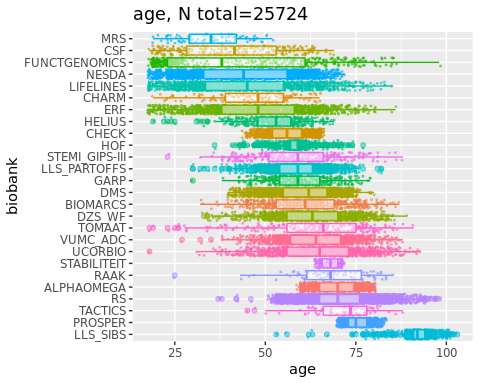 | Binarized variable  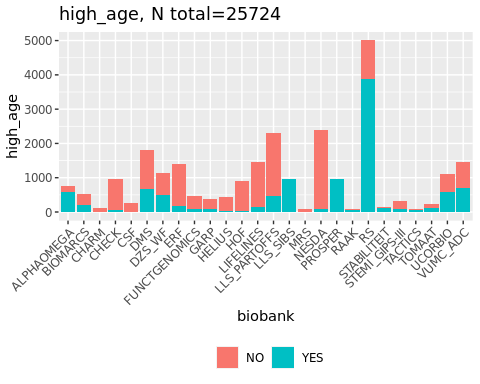 |
| --- | --- |
| Binarized variable  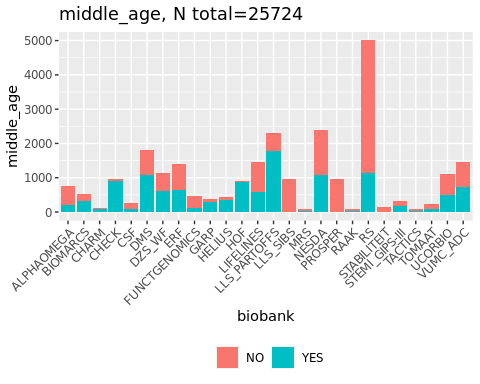 | Binarized variable  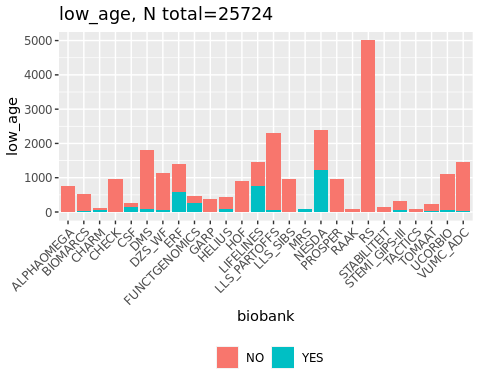 |

Obesity

| Original variable  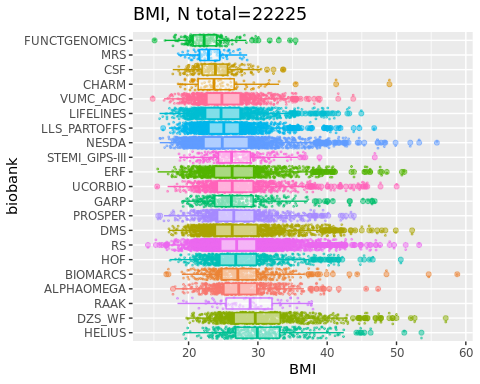 | Original variable  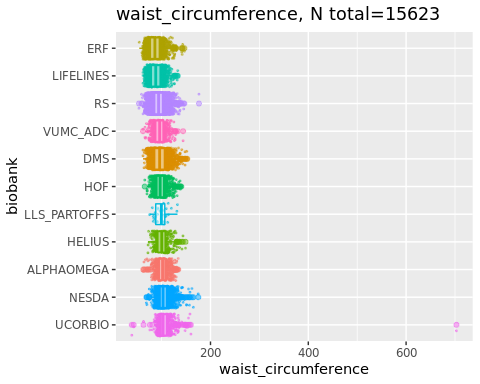 |
| --- | --- |
| Binarized variable  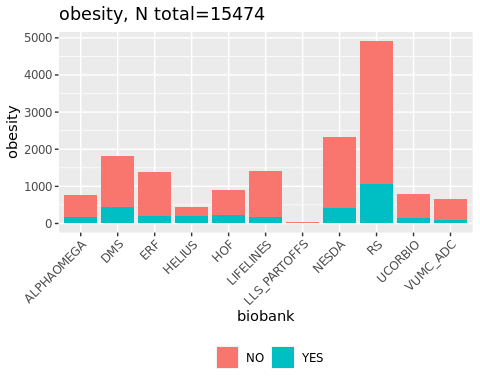 |  |

High pressure

| Original variable  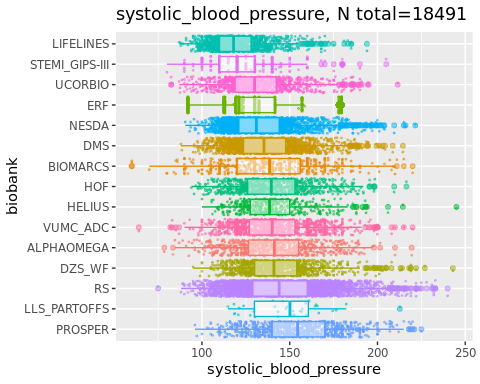 | Original variable  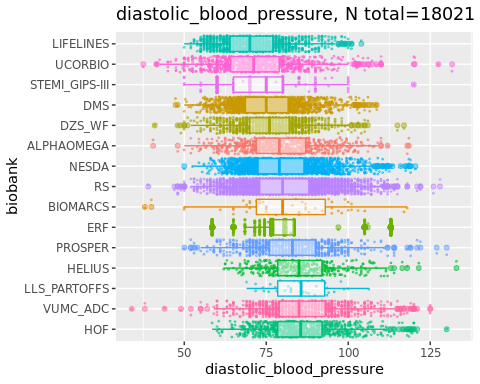 |
| --- | --- |
| Binarized variable  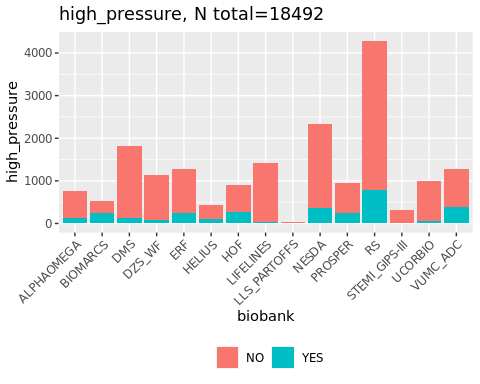 |  |

ln_hscrp

| Original variable  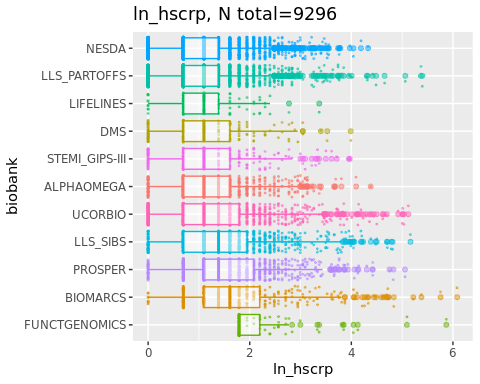 | Binarized variable  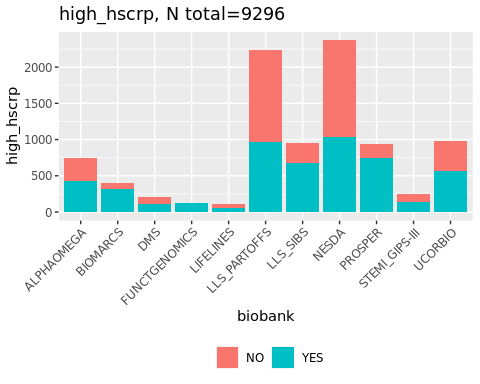 |
| --- | --- |

Tryglicerides

| Original variable  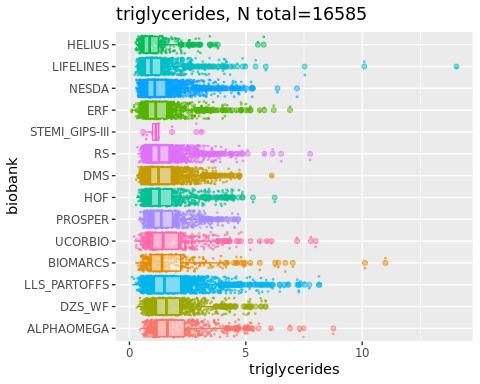 | Binarized variable  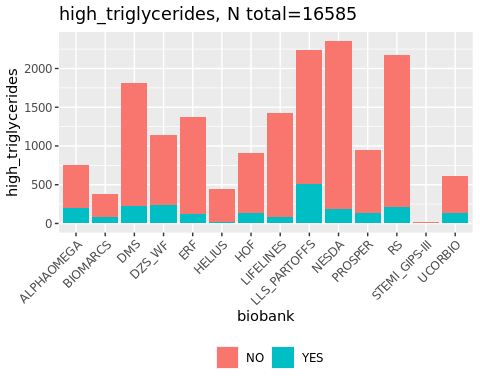 |
| --- | --- |

**ldl_cholesterol**

| Original variable  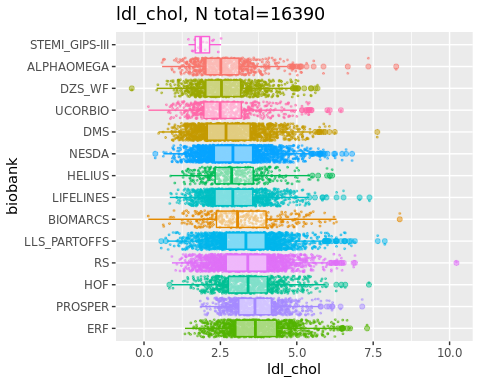 | Binarized variable  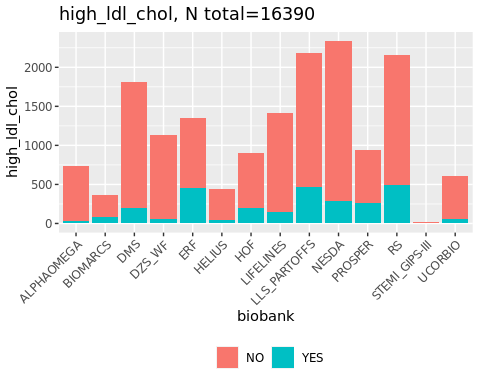 |
| --- | --- |

hdl cholesterol

| Original variable  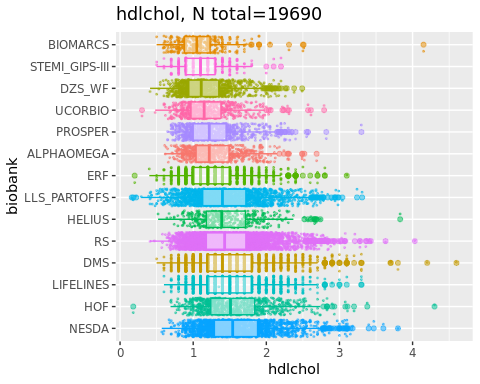 | Binarized variable  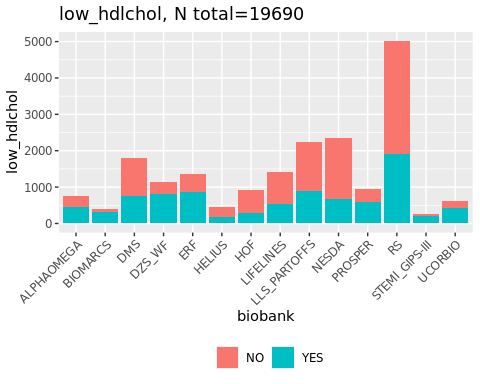 |
| --- | --- |

total cholesterol

| Original variable  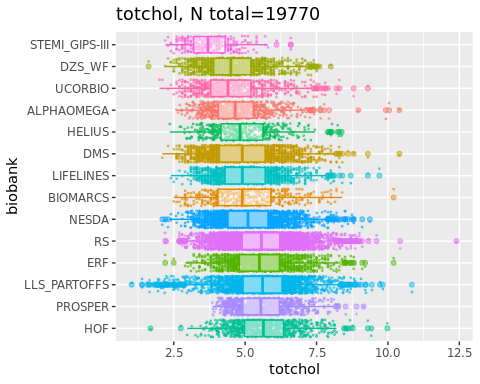 | Binarized variable  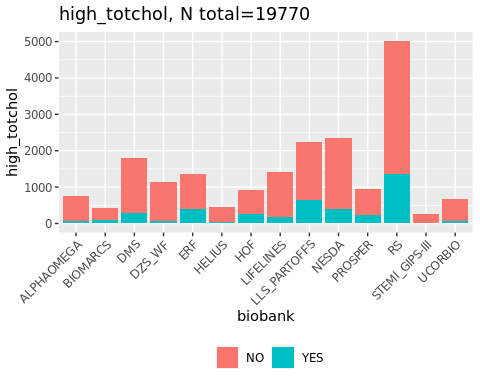 |
| --- | --- |

eGFR

| Original variable  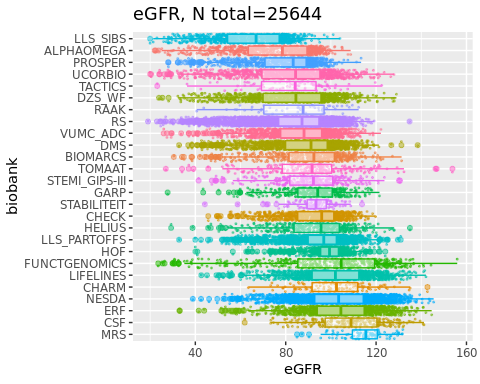 | Binarized variable  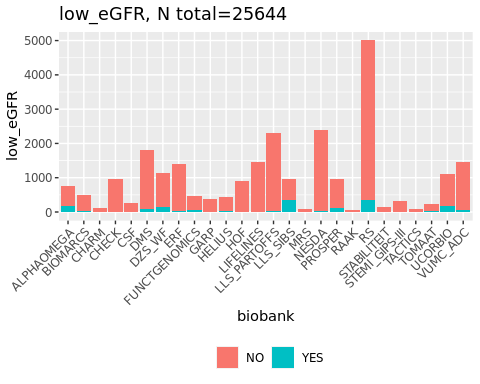 |
| --- | --- |

Hgb

| Original variable  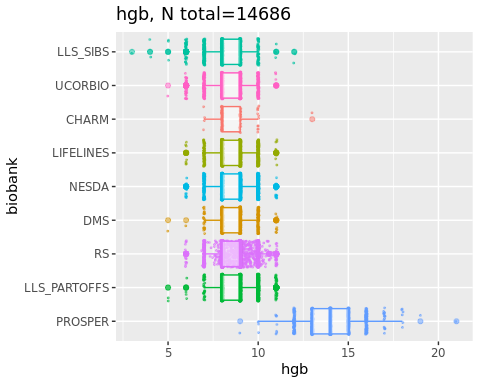 | Binarized variable  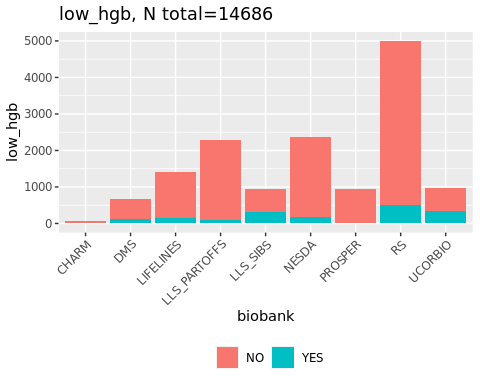 |
| --- | --- |

Wbc

| Original variable  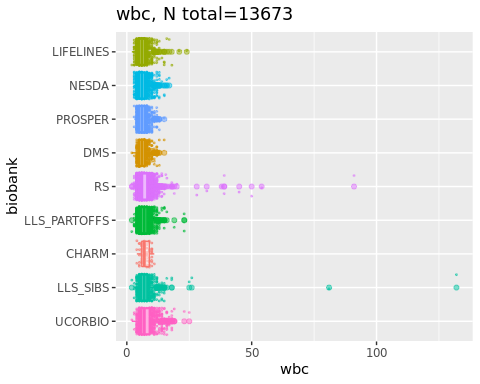 | Binarized variable  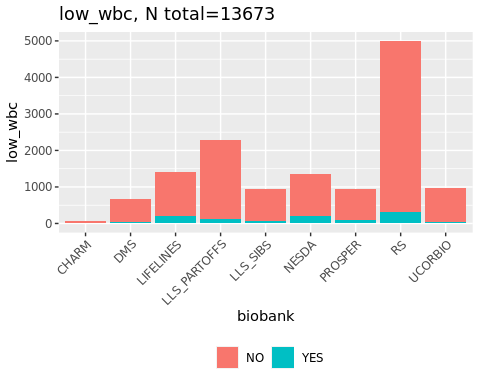 |
| --- | --- |

Session info

R version 4.0.3 (2020-10-10) Platform: x86_64-conda-linux-gnu (64-bit) Running under: Ubuntu 18.04.5 LTS

Matrix products: default BLAS/LAPACK: /etc/miniconda/lib/libopenblasp-r0.3.12.so

locale: [1] LC_CTYPE=C.UTF-8 LC_NUMERIC=C LC_TIME=C.UTF-8
[4] LC_COLLATE=C.UTF-8 LC_MONETARY=C.UTF-8 LC_MESSAGES=C.UTF-8
[7] LC_PAPER=C.UTF-8 LC_NAME=C LC_ADDRESS=C
[10] LC_TELEPHONE=C LC_MEASUREMENT=C.UTF-8 LC_IDENTIFICATION=C

attached base packages: [1] parallel grid stats graphics grDevices utils datasets [8] methods base

other attached packages: [1] dplyr_1.0.5 broom_0.7.6
[3] ggpmisc_0.3.1 pcaMethods_1.82.0
[5] Biobase_2.50.0 BiocGenerics_0.36.0
[7] plyr_1.8.6 formattable_0.2.1
[9] RColorBrewer_1.1-2 gtools_3.8.2
[11] pheatmap_1.0.12 PerformanceAnalytics_2.0.4 [13] xts_0.12.1 zoo_1.8-9
[15] pROC_1.17.0.1 gridExtra_2.3
[17] plotmo_3.6.0 TeachingDemos_2.12
[19] plotrix_3.8-1 Formula_1.2-4
[21] e1071_1.7-6 glmnet_2.0-18
[23] Matrix_1.3-2 caret_6.0-86
[25] lattice_0.20-44 foreach_1.5.1
[27] ggcorrplot_0.1.3 ggplot2_3.3.3
[29] data.table_1.14.0 matrixStats_0.58.0
[31] ggsci_2.9 edgeR_3.32.1
[33] limma_3.46.0 knitr_1.31

loaded via a namespace (and not attached): [1] nlme_3.1-152 lubridate_1.7.10 tools_4.0.3
[4] backports_1.2.1 utf8_1.2.1 R6_2.5.0
[7] rpart_4.1-15 DBI_1.1.1 colorspace_2.0-0
[10] nnet_7.3-15 withr_2.4.2 tidyselect_1.1.1
[13] compiler_4.0.3 labeling_0.4.2 scales_1.1.1
[16] quadprog_1.5-8 proxy_0.4-25 stringr_1.4.0
[19] digest_0.6.27 rmarkdown_2.7 pkgconfig_2.0.3
[22] htmltools_0.5.1.1 highr_0.9 htmlwidgets_1.5.3
[25] rlang_0.4.11 generics_0.1.0 farver_2.1.0
[28] ModelMetrics_1.2.2.2 magrittr_2.0.1 Rcpp_1.0.6
[31] munsell_0.5.0 fansi_0.4.2 lifecycle_1.0.0
[34] stringi_1.5.3 yaml_2.2.1 MASS_7.3-53.1
[37] recipes_0.1.16 crayon_1.4.1 splines_4.0.3
[40] locfit_1.5-9.4 pillar_1.6.0 reshape2_1.4.4
[43] codetools_0.2-18 stats4_4.0.3 glue_1.4.2
[46] evaluate_0.14 vctrs_0.3.8 gtable_0.3.0
[49] purrr_0.3.4 tidyr_1.1.3 assertthat_0.2.1
[52] xfun_0.20 gower_0.2.2 prodlim_2019.11.13
[55] class_7.3-18 survival_3.2-11 timeDate_3043.102
[58] tibble_3.1.1 iterators_1.0.13 lava_1.6.9
[61] ellipsis_0.3.2 ipred_0.9-11
